# CRISPR-Cas9-directed gene tagging using a single integrase-defective lentiviral vector carrying a transposase-based Cas9 off switch

**DOI:** 10.1016/j.omtn.2022.08.005

**Published:** 2022-08-04

**Authors:** Emil Aagaard Thomsen, Kristian Alsbjerg Skipper, Sofie Andersen, Didde Haslund, Thomas Wisbech Skov, Jacob Giehm Mikkelsen

**Affiliations:** 1 Department of Biomedicine, HEALTH, Aarhus University, Høegh-Guldbergs Gade 10, 8000 Aarhus C, Denmark

**Keywords:** MT: Delivery Strategies, lentivirus, AAV, CRISPR-Cas9, piggyBac, protein delivery, DNA transposon, HDR, Donor template, IDLV, Gene tagging

## Abstract

Locus-directed DNA cleavage induced by the CRISPR-Cas9 system triggers DNA repair mechanisms allowing gene repair or targeted insertion of foreign DNA. For gene insertion to be successful, availability of a homologous donor template needs to be timed with cleavage of the DNA by the Cas9 endonuclease guided by a target-specific single guide RNA (sgRNA). We present a novel approach for targeted gene insertion based on a single integrase-defective lentiviral vector (IDLV) carrying a Cas9 off switch. Gene insertion using this approach benefits from transposon-based stable Cas9 expression, which is switched off by excision-only transposase protein co-delivered in IDLV particles carrying a combined sgRNA/donor vector. This one-vector approach supports potent (up to >80%) knockin of a full-length EGFP gene sequence. This traceless cell engineering method benefits from high stable levels of Cas9, timed intracellular availability of the molecular tools, and a built-in feature to turn off Cas9 expression after DNA cleavage. The simple technique is based on transduction with a single IDLV, which holds the capacity to transfer larger donor templates, allowing robust gene knockin or tagging of genes in a single step.

## Graphical abstract

**Figure:**
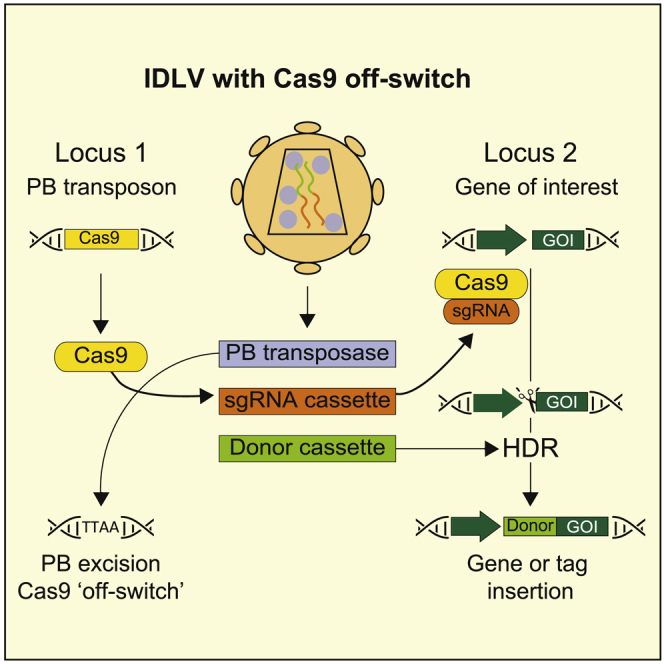

## Teaser

Thomsen et al. present a one-step approach to traceless cell engineering by targeted gene or gene tag insertion, accomplished by a single integrase-defective lentiviral vector (IDLV) carrying a built-in transposase-based Cas9 off switch. This one-vector approach utilizes stable Cas9 expression and homology-directed repair to support potent knockin of genes including larger donor templates.

## Introduction

With the CRISPR-Cas wave still moving at full speed, attention remains focused on new developments that can optimize and deliver the CRISPR-Cas toolbox for a wide range of applications.^1^ Discovering and developing new RNA-guided endonucleases and employing these in new, innovative ways to optimize cutting efficiency, reduce off-target cleavage, and repurpose the system beyond genome editing have pushed the technology forward at an unprecedented pace.^2^, ^3^, ^4^ Although the CRISPR-Cas9 system is continuously being optimized, the ability to standardize efficient homology-directed repair (HDR) using a co-delivered donor template remains a prominent challenge for introducing small nucleic acid changes or integrating larger transgenic elements. Intervention by CRISPR-Cas9 can be effectively achieved in cell lines by plasmid-based expression of the CRISPR-Cas9 components (Cas9 endonuclease and single guide RNA, sgRNA),^5^, ^6^, ^7^ but nucleofection of recombinant Cas9 protein complexed with a synthetic sgRNA as a ribonucleoprotein (RNP) is attracting increasing attention for applications in cell lines and primary cells.^2^^,^^8^, ^9^, ^10^ By delivering RNP complexes directly to the target cells rather than as plasmid DNA or *in-vitro*-transcribed mRNA, targeted double-stranded breaks (DSBs) are introduced almost immediately, and the RNP complex is rapidly degraded.^11^ This short boost of exposure to Cas9 and sgRNA ensures high on-target DNA cutting and supposedly minimizes aberrant off-target cleavage. In addition to RNPs, we and others have sought to develop alternative delivery approaches (e.g., methods based on virus-based protein delivery) for minimizing exposure to the active Cas9-sgRNA complex.^12^, ^13^, ^14^, ^15^ Independent of the Cas9/sgRNA administration approach, the donor template can be supplied in *trans* as naked DNA^9^^,^^16^ or by packaging the donor template into a viral vector, typically a lentiviral vector or a vector based on an adeno-associated virus (AAV).^8^^,^^17^ Nevertheless, HDR-mediated knockin remains challenging, and for some applications, effective knockin may be affected by restricted administration of small donor templates.^12^^,^^15^ Efficacy of a knockin genome editing approach based on HDR relies on synchronicity between DNA cleavage and donor DNA availability. If the donor repair template is not available in the immediate vicinity of the DNA cleavage site in a short time frame after cutting, then the break is prone to be repaired by nonhomologous end joining (NHEJ) and not by HDR.^18^ Timing may therefore be essential for optimal efficacy. Attempts to achieve CRISPR-Cas9-directed DNA cutting and concomitant repair are most often driven by the idea of combining a high intracellular concentration of Cas9/sgRNA complexes with estimates of when to deliver the donor sequence for optimal efficacy.^19^^,^^20^ In many cases, this involves including several delivery strategies to obtain timed delivery of the DNA-cutting entity and the repair template. To circumvent timing issues and alleviate the fast repair by NHEJ, various inhibitors suppressing NHEJ have been studied in efforts to favor repair by HDR.^18^^,^^21^^,^^22^

Vectors derived from DNA transposons, typically Sleeping Beauty or piggyBac, offer an alternative, nonviral approach for introducing transgenes into genomes. Upon excision from a genomic harbor, the piggyBac DNA transposon does not leave a genetic footprint,^23^ which makes this technology relevant for applications where inserted transgenes need to be removed seamlessly from the genome. Such uses include insertion and removal of genes encoding reprogramming factors for production of induced pluripotent stem cells (iPSCs),^24^ removal of selection cassettes included in HDR donor templates,^25^ and generation of Cas9-expressing cells in which Cas9 expression can be shut down by excision of a Cas9-encoding gene cassette.^12^ To facilitate excision only without risking re-insertion of the excised transposon in another locus, the piggyBac transposase was previously successfully modified by alteration of three amino acids, rendering it integration incompetent without disturbing the capacity to excise.^26^ By nucleofecting this transposase variant into iPSCs, Wang et al.^12^ demonstrated the potential of utilizing the excision-only transposase variant for switching off Cas9 expression.

We have previously utilized HIV-derived lentiviral particles as vessels for direct delivery of proteins of nonviral origin. Fusing such heterologous proteins to the N terminus of the Gag/GagPol polypeptide leads to effective protein incorporation in virus particles, resulting in release and activity of the protein in cells exposed to the viral particles. Utilizing such protein ferries, we have delivered the hyperactive piggyBac transposase hyPBase,^27^ zinc-finger nucleases (ZFNs),^28^^,^^29^ TAL-effector nucleases (TALENs),^29^ and Cas9 originating from *Streptococcus pyogenes*.^13^ Most recently, we expanded on this design and fused the hyperactive piggyBac transposase hyPBase^30^ to the C terminus of the GagPol polypeptide.^31^ In contrast to our previous observations with N-terminally tagged GagPol polypeptides, this design allowed co-packaging and transfer of a transposon donor within an integrase-deficient transfer vector without the need of titrating in unmodified GagPol polypeptide during lentiviral vector production.^32^ Relative to delivery methods based on transfection of plasmid DNA or *in-vitro*-transcribed mRNA, we demonstrated that direct protein delivery by lentiviral protein transduction resulted in time-restricted protein activity within the target cells without compromising efficacy.^31^

In the present work, we set out to utilize lentiviral transposase protein delivery to switch off stable expression of the Cas9 endonuclease by excising a Cas9-expressing transposon from the genome. This feature allowed potent targeted DNA cleavage upon sgRNA delivery, leading to effective targeted knockin of gene tags delivered as HDR repair templates on vector RNA in transposase-loaded lentiviral vectors. This approach ensures that Cas9-sgRNA complexes can only be formed when the donor is also available upon reverse transcription and aims to synchronize DNA cutting after Cas9-sgRNA complex formation with immediate availability of the appropriate donor sequence. Our findings demonstrate traceless generation of knockin cell lines and offer a robust approach to facilitate efficient CRISPR-Cas9-directed targeted gene insertion.

## Results

### Genomic excision of the piggyBac transposon by hyPB^Exc+^ excision-only transposase delivered in lentivirus-derived nanoparticles (LVNPs)

**Figure 1:**
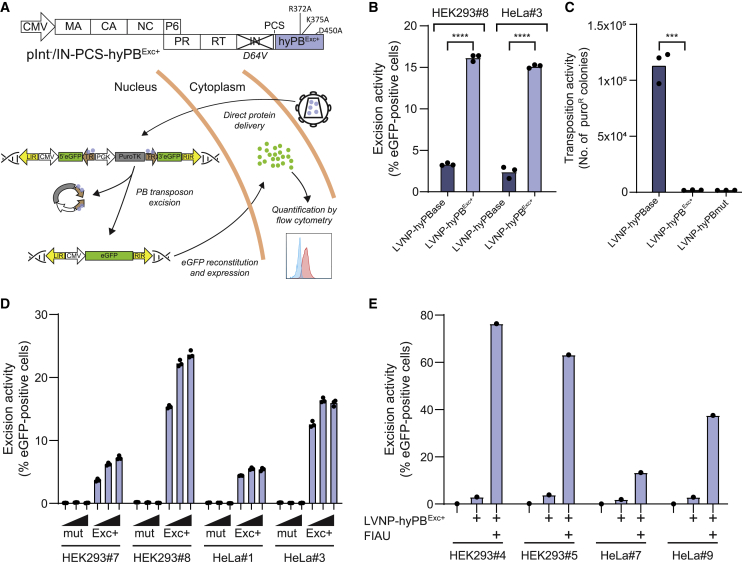
Protein transduction of hyPB^Exc+^ efficiently excises genomically integrated transposons (A) Schematic of the GagPol-hyPB^Exc+^ fusion vector and an overall outline of the reporter system used for evaluating hyPB^Exc+^-mediated genomic excision. (B) LVNP-hyPB^Exc+^ protein delivery noticeably increases genomic transposon excision in HeLa and HEK293 reporter cell lines. ∗∗∗∗p < 0.0001 (unpaired t test). (C) Comparison of PB transposition efficiency of hyPB protein variants after delivery by lentiviral protein transduction and transfection of pPBT/PGK-Puro. ∗∗∗p < 0.001 (unpaired t test). (D) Repeated LVNP dosing only marginally increases transposon excision rates. HeLa and HEK293 reporter cell lines were transduced with hyPBmut- or hyPB^Exc+^-loaded LVNPs on 3 consecutive days. Excision rates were quantified by flow cytometry after single, double, and triple LVNP delivery, as indicated by black triangles. (E) Enrichment of transposon excision by negative FIAU selection. Experiments were performed in biological triplicates (individual wells); bars represent mean, with dots corresponding to individual replicates.

To facilitate potent excision of piggyBac DNA transposons from their genomic harbor,^33^ we first generated an excision-only hyPBase transposase variant (hyPB^Exc+^) based on introduction of three amino acid changes (R372A, K375A, and D450A; Figure S1), which are known to restrict the transposon insertion capacity of the iPB7 transposase without affecting the ability of the enzyme to excise transposons.^26^ Using an EGFP reporter construct (Figure S1A), solid hyPB^Exc+^-directed transposon excision from transfected plasmid DNA (Figure S1B) and genomic DNA (Figures S1C and S1D) was verified in cells transfected with a hyPB^Exc+^ expression plasmid. We also verified the restricted capacity of hyPB^Exc+^ to support DNA transposition relative to the normal hyPBase (Figure S1E), demonstrating that hyPB^Exc+^ was excision competent and insertion incompetent. To explore direct delivery of hyPB^Exc+^ protein using LVNPs, we fused the hyPB^Exc+^ sequence to the integrase gene in the 3′-terminal end of the GagPol reading frame (Figure 1A).^31^ We then transduced HEK293 and HeLa reporter clones with LVNP-hyPB^Exc+^ and LVNP-hyPBase (see Materials and methods for an overview of nomenclature for LVNPs, integrase-deficient lentiviral vectors [IDLVs], and IDLVs carrying foreign protein; Figure 1A). Four days after LVNP-hyPB^Exc+^ delivery, EGFP reconstitution had occurred in more than 15% of the cells in both clones, which was substantially higher than in cells treated with LVNP-hyPBase, resulting in only 2%–3% EGFP-positive cells (Figure 1B). We observed potent DNA transposition after LVNP-based delivery of hyPBase, resulting in 1.1 × 10^5^ puromycin-resistant colonies (SD = 1.5 × 10^3^ colonies), whereas delivery of hyPBmut did not result in transposition (Figure 1C). Contrary to plasmid-based delivery of hyPB^Exc+^ (Figure S1E), LVNP-mediated hyPB^Exc+^ delivery did not induce colony formation (Figure 1C). These data demonstrate that LVNP-delivered hyPB^Exc+^ effectively excises transposons but does not support genomic insertion of transposons.

To further boost transposon excision rates, we treated HeLa and HEK293 reporter cells with LVNP-hyPBmut or LVNP-hyPB^Exc+^ on three consecutive days. For a total of four clones, we found that repeated LVNP dosing led to increasing levels of EGFP, as measured by flow cytometry (Figure 1D). However, the effect was marginal, and repeated administration increased the number of EGFP-positive cells from 15% to 24% at most, suggesting that transposons in at least part of the cell population were not excisable. To circumvent this issue, we applied Fialuridine (FIAU) selection to four clones (HEK293#4, HEK293#5, HeLa#7, and HeLa#9) representing single- and multi-copy clones of each cell line (Figure S2) with a low level of excision after treatment with LVNP-hyPB^Exc+^. For three of four clones, this resulted in a dramatic increase in the percentage of EGFP-positive cells (Figure 1E). Because all untreated cells died in the presence of FIAU (because of expression of the puroTK cassette encoding puromycin N-acetyltransferase fused to a truncated HSV-1 thymidine kinase), these findings supported the notion that the cell population was strongly enriched for piggyBac transposon-deficient cells driven by the LVNP-hyPB^Exc+^-mediated transposon excision combined with FIAU selection. Seamless transposon excision in FIAU-resistant clones was confirmed by Sanger sequencing (Figure S3).

### Effective CRISPR-Cas9-directed knockout in cells carrying the hyPB^Exc+^-excisable Cas9 expression cassette

**Figure 2:**
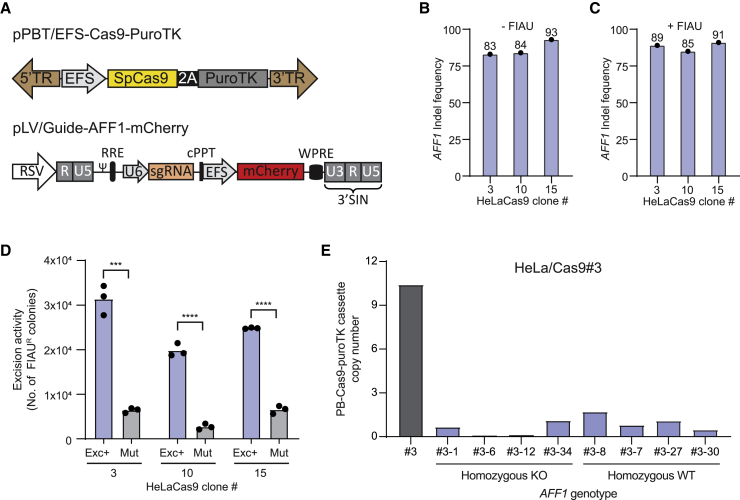
IDLV-mediated co-delivery of sgRNA and hyPB^Exc+^ protein for highly efficient generation of Cas9-negative knockout cell lines (A) Schematic of the vectors used for creation of Cas9-PuroTK-expressing cell lines (pPBT/EFS-Cas9-PuroTK) and for co-delivering sgRNA and hyPB^Exc+^ protein (pLV/guide-AFF1-mCherry). (B and C) AFF1 indel formation was quantified by ICE in HeLa PB/Cas9-PuroTK cell lines transduced with IDLV-hyPB^Exc+^/sgRNA.AFF1 before FIAU (B) and after enrichment of PB/Cas9-PuroTK transposon excision by negative selection with FIAU (C). (D) HeLa cell lines carrying different copy numbers of the PB/Cas9-PuroTK transposon were treated with IDLV-hyPB^Exc+^ co-delivering hyPB^Exc+^ protein and a sgRNA targeting *AFF1* (AFF1) or LVNPs delivering hyPBmut protein (mut). Enrichment was quantified by colony formation. ∗∗∗p < 0.0005, ∗∗∗∗p < 0.0005 (unpaired t test). (E) Quantification of transposon excision in single clones isolated from HeLa PB/Cas9-PuroTK cells transduced with the IDLV-hyPB^Exc+^/sgRNA.AFF1; *AFF1* genotype was assessed by ICE analysis and excision of transposon with ddPCR. Experiments were performed in biological triplicates (individual wells); bars represent mean, with dots corresponding to individual replicates.

To ensure high availability of Cas9 during genome editing and to restrict prolonged Cas9 production, we designed a piggyBac transposon vector (pPBT/EFS-Cas9-PuroTK) carrying a Cas9 expression cassette fused to puroTK via a 2A peptide sequence (Figure 2A). This configuration allowed stable Cas9 expression to be switched off by footprint-free excision of the transposon, leading to its loss during cell growth. The transposon was inserted into genomic DNA of HeLa cells by standard transfection methods, and copy numbers were determined in puromycin-resistant colonies using droplet digital PCR (ddPCR) (Figure S4). We proceeded with three HeLaCas9 clones, two low-copy clones (clones #10 and #15 carrying 2 and 3 copies, respectively), and one high-copy clone (clone #3 carrying 10 copies). Each of the three clones was then transduced with IDLV-hyPB^Exc+^/sgAFF1-mCherry (containing LV/guide-mCherry vector RNA with the sequence of an sgRNA targeting the *AFF1* gene; Figure 2A). Despite the difference in copy numbers between clones, all three Cas9-expressing clones supported high insertion or deletion (indel) rates above 80%, as measured by sequencing and ICE analysis 4 days after transduction (Figure 2B). These rates matched indel rates we typically see in cells transduced with lentiviral vectors expressing Cas9 and a potent sgRNA.^34^ FIAU selection did not further increase the indel rates (Figure 2C), although treatment with IDLV-hyPB^Exc+^/sgAFF1-mCherry markedly increased the number of FIAU-resistant colonies relative to IDLV-hyPBmut/sgAFF1-mCherry (Figure 2D), suggesting that *AFF1*-targeted endonuclease activity was at its maximum in cells showing hyPB^Exc+^-directed removal of the Cas9-expressing transposon.

Next we generated 44 FIAU-resistant clones derived from HeLaCas9#3 transduced with IDLV-hyPB^Exc+^/sgAFF1-mCherry and examined the *AFF1* locus. Thirty-six of 44 clones (81.8%) were homozygous for the knockout allele, 2 clones (4.5%) were heterozygous, and the remaining 6 clones (13.6%) were homozygous for the wild-type allele (Figure S5). Across the 44 clones, the total fraction of edited alleles was 84%, mirroring the indel rate determined for the unselected clone (Figures 2B and 2C). To explore the relationship between indel formation and transposon excision events, we quantified transposon excision by ddPCR in 8 clones: 4 that were homozygous for the wild-type allele and 4 that were homozygous for the knockout mutation (Figure 2E). In both groups of clones, all originally carrying 10 copies, we observed robust excision resulting in clones with 1 or 2 copies and clones with all transposons removed. These data demonstrate strong excision capacity and argue that events of indel formation occur alongside transposon excision after delivery of the sgRNA expression cassette and hyPB^Exc+^ protein in LVNPs.

### Simultaneous HDR-dependent knockin and Cas9 switch-off after IDLV delivery of the sgRNA cassette, donor sequence, and excision-only transposase protein

**Figure 3:**
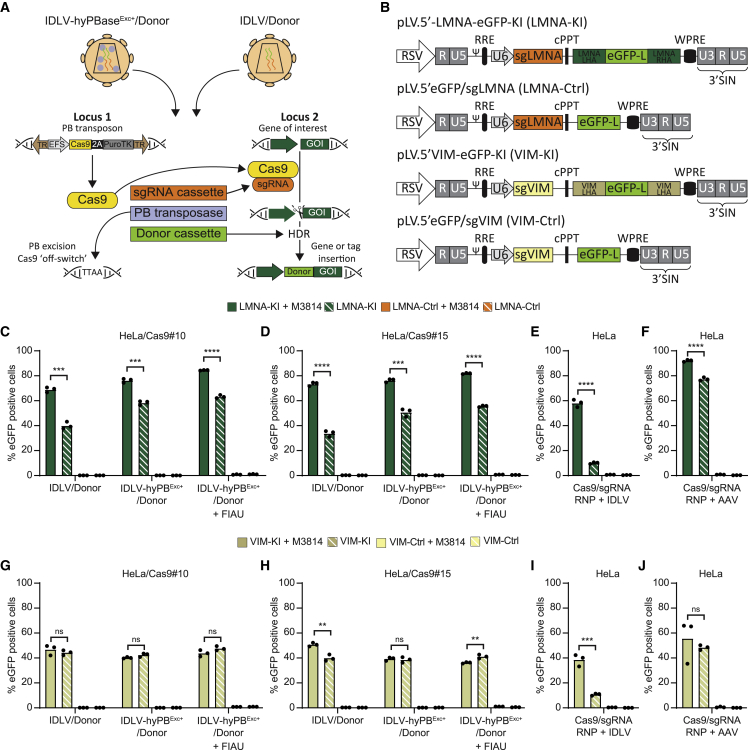
Efficient, traceless, and Cas9-mediated HDR-based EGFP tagging of endogenous proteins (A) Schematic of the donor vector co-delivered with hyPB^Exc+^ protein. (B) Vector schematics of KI donor vectors with homology arms and sgRNA or without arms (Ctrl) and sgRNA. (C–F) EGFP insertion into the *LMNA* locus. (G–J) EGFP insertion into the *VIM* locus. HeLaCas9#10 cells (C and G) and HeLaCas9#15 cells (D and H) were transduced with IDLV/donor or IDLV-hyPB^Exc+^/donor corresponding to 40 ng P24. HDR-based EGFP tagging was quantified by flow cytometry. Cells were transduced with 40 ng P24 of IDLV/donor. (E and I) or AAV/donor at an MOI of 1 × 10^5^. (F and J) and immediately thereafter nucleofected with Cas9/sgRNA. Experiments were performed in biological triplicates (individual wells); bars represent mean, with dots corresponding to individual replicates. ∗∗p < 0.005, ∗∗∗p < 0.0005, ∗∗∗∗p < 0.0005 (unpaired t test).

Timing of the availability of the Cas9-sgRNA complex with donor accessibility within a certain time window is crucial for effective CRISPR-Cas9-directed DNA repair or gene insertion by HDR. We hypothesized that timing could be achieved by delivering vector RNA carrying the sgRNA-encoding cassette and an HDR donor sequence to cells showing high stable expression of Cas9. We reasoned that Cas9 expression at the same time could be switched-off by co-delivering hyPB^Exc+^ in the IDLVs, facilitating genomic excision of the transposon-based Cas9 expression cassette (Figure 3A). To evaluate the HDR capacity of this approach, we tagged the genes encoding Lamin A/C and vimentin (*LMNA* and *VIM*, respectively) with a full-length EGFP gene cassette, allowing fluorescent fusion proteins to be produced in vector-treated cells. HDR has been exploited previously to tag *LMNA* and *VIM* with a 48-bp fragment of the EGFP gene (in cells containing the remaining part of EGFP),^35^ but here we aimed to insert the entire 735-bp EGFP-coding sequence. Initially, we engineered two donor vectors (Figure 3B) carrying the EGFP gene flanked by 360-bp left and right homology arms (LHAs and RHAs, respectively), allowing homologous recombination into the *LMNA* and *VIM* genes. The corresponding donor vectors without homology arms served as controls. In all four vectors, sgRNAs targeting the two genes were expressed from an expression cassette driven by the U6 promoter situated upstream of the HDR donor sequence (Figure 3B). We set out to determine EGFP knockin rates HeLaCas9 clones #10 and #15, carrying 2 and 3 copies, respectively, of the Cas9-encoding piggyBac DNA transposon (Figure S4). In both clones, transduction with regular IDLVs or hyPB^Exc+^-loaded IDLVs carrying the LMNA sgRNA cassette and the donor cassette containing the EGFP tag flanked by homology arms (referred to as LMNA knockin [KI]) provided robust KI rates, resulting in up to 58.4% (SD = 1.7%) EGFP-positive cells (among cells treated with hyPB^Exc+^-loaded IDLVs) when DNA repair by NHEJ was not restricted (Figures 3C and 3D; gating strategy for flow cytometry in Figure S6). The insertion rates were further increased in the presence of the PRKDC inhibitor M3814, which has been shown previously to improve DNA insertion by HDR,^18^^,^^21^ resulting in up to 68.9% (SD = 2.0%) and 76.3% (SD = 1.4%) EGFP-positive cells among cells treated with IDLVs (with and without hyPB^Exc+^, respectively). By selecting for cells in which the transposon was excised (by applying FIAU), rates for EGFP insertion into the *LMNA* gene were improved even further, reaching more than 84.5% (SD = 0.23%) and 81.9% (SD = 0.45%) EGFP-positive cells in HeLaCas9 clones #10 and #15, respectively.

### Insertion rates are higher than or comparable with state-of-the-art gene insertion approaches

For comparison, we aimed to determine KI rates using state-of-the-art strategies based on co-delivery of Cas9/sgRNA and the donor sequence. First, a basic plasmid-based approach based on co-transfection of two plasmids in HeLa cells, one carrying a Cas9 expression cassette and one carrying the sgRNA expression cassette plus the donor sequence, resulted only in very low levels of cells with stable EGFP expression (approximately 1% after 14 days; Figures S7A–S7C). We also introduced the EGFP tag in normal HeLa cells based on nucleofection with Cas9/sgRNA RNP complexes combined with IDLV- or AAV-based delivery of the donor sequence (with or without LMNA homology arms). Using IDLV as the source of the donor sequence, this approach resulted in 58.1% (SD = 2.9%) EGFP-positive cells in the presence of M3814 (Figure 3E). However, in the absence of M3814, the level of EGFP-positive cells was markedly reduced (10.0%, SD = 0.7%), suggesting that timing of DNA cleavage and donor availability was achieved only by inhibiting repair by NHEJ. In a similar setup using AAV6 as a source of the donor DNA, effective KI was achieved, resulting in 77.3% (SD = 1.32%) and 92.1% (SD = 0,83%) EGFP-positive cells in the absence and presence of M3814, respectively (Figure 3F). With IDLV and AAV6 donors, EGFP-positive cells were not observed with donors without homology arms (Figures S7D–S7F).

In a similar set of experiments, we measured EGFP insertion rates in *VIM*. For this locus, we obtained up to 43.8% (SD = 2.5%) and 36.1% (SD = 0.58%) EGFP-positive cells in Cas9-expressing clones HeLa#10 and HeLa#15 (Figures 3G and 3H). Interestingly, an effect of M3814 was not evident for insertion into *VIM*, although tagging with EGFP was clearly dependent on homology arms and repair by HDR. As for insertion into *LMNA*, combined administration of Cas9/sgRNA RNPs and an IDLV donor effectively resulted in EGFP insertion only in the presence of M3814 (Figure 3I). In contrast, M3814 did not alter the insertion rate when AAV served as a donor (Figure 3J), and combined delivery of Cas9/sgRNA RNPs and an AAV donor resulted in levels of EGFP tagging that were comparable with IDLVs used in combination with stable Cas9 expression switched off during the insertion process (Figures 3G and 3H). Our data suggest that an IDLV-based KI approach relying on stable expression of Cas9 (combined with excision of the cassette using hyPB^Exc+^ protein delivery) performed markedly better than an approach based on co-delivery of the IDLV donor and Cas9/sgRNA RNPs. Using AAV as the donor, the efficacy was higher, suggesting that DNA intermediates using this donor were immediately available for DNA repair by HDR. Also, we showed by ddPCR that levels of donor DNA were in general higher in cells treated with AAV donors than in cells treated with IDLV donors (Figure S7G). We conclude that the described approach, based on stable Cas9 expression and delivery of sgRNA and donor cassettes, resulted in levels of KI that were higher than or comparable with state-of-the-art methods.

To enrich the cell population for cells in which the Cas9-encoding transposon had been excised, we exposed LVNP-hyPB^Exc+^-treated cells with FIAU (Figures 3C, 3D, 3G, and 3H). This treatment slightly increased the number of EGFP-positive cells only in the case of *LMNA*, arguing that EGFP KI was, in most cells, accompanied by transposon excision by co-delivered hyPB^Exc+^. These findings demonstrated production of stably expressing EGFP-positive cells by HDR with immediate shutoff of Cas9 expression because of excision and loss of transposons as the original source of Cas9, resulting in cells with EGFP expression and restricted SpCas9 expression.

### Subcellular localization of tagged proteins indicative of potent targeted gene KI

**Figure 4:**
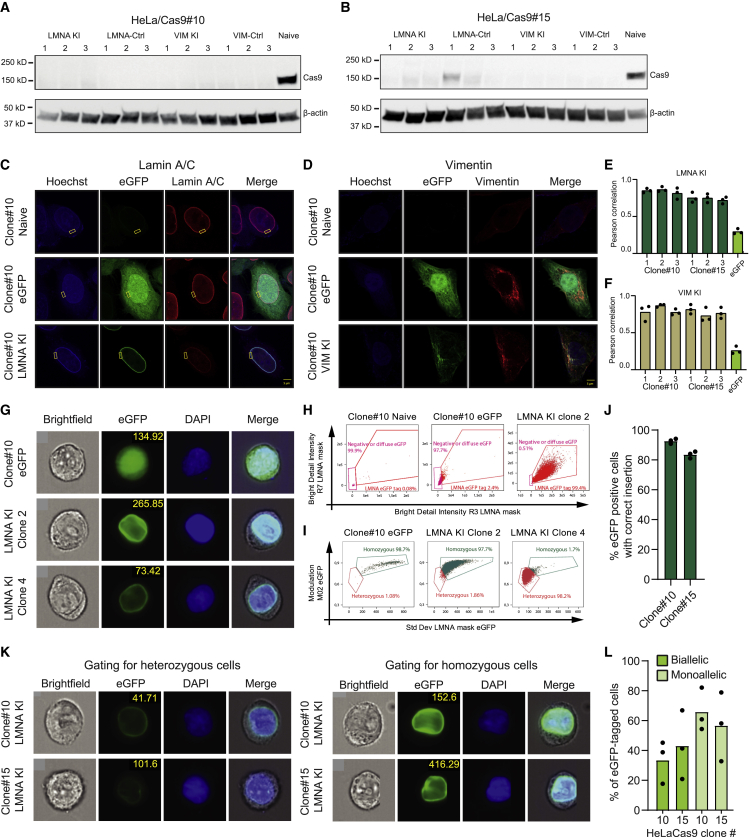
Quantification of targeted EGFP KI in *LMNA* and *VIM* (A and B) Verification of transposon excision by western blot using Cas9 antibody after FIAU selection in HeLa PB/Cas9-PuroTK clone #10 (A) and clone #15 (B). (C and D) Representative images from confocal microscopy of cells expressing EGFP-tagged LMNA (C) and VIM (D) with antibody staining for the corresponding tagged protein. Scale bars, 5 μM. (E and F) Pearson correlation analysis of EGFP (tag) and Alexa Fluor 647 (antibody) intensity in each pixel, with the ROI focusing on the nuclear membrane for LMNA (E) and the cytoplasm for VIM (F). (G) Representative images of the samples used for ImageStream analysis. Data are shown for Hela/Cas9#10 expressing EGFP and two LMNA KI clones. (H) ImageStream gating strategy for separation of LMNA EGFP-tagged cells and cells with a diffuse EGFP expression pattern. Discrimination of cells is based on a nuclear confined mask (LMNA mask). (I) ImageStream gating strategy for separation of homozygous and heterozygous LMNA EGFP-tagged populations. (J) ImageStream-based quantification of correct LMNA EGFP-tagged cells and distribution of homozygous and heterozygous KI events. (K) Representative images of heterozygous cells (top row) and homozygous cells (bottom row) for HeLaCas9#10 and HeLaCas9#15 LMNA KI cells. (L) ImageStream-based quantification of bi-allelic and mono-allelic EGFP-tagged LMNA loci. Experiments were performed in biological triplicates (individual wells); bars represent mean, with dots corresponding to individual replicates.

Next we investigated transposon excision by analyzing expression of Cas9 protein using western blotting (Figures 4A, 4B, S8, and S9) and found that expression of Cas9 was switched off in the majority of FIAU-treated cells. Copy number analysis by ddPCR demonstrated effective transposon excision (Figure S10), suggesting that only a small fraction of the treated cells still contained a transposon cassette. It is unclear whether these few remaining transposons were transcriptionally active and gave rise to traits of transposase expression observed by western blotting (Figure 4B) or were potentially silenced. Second, we studied the intracellular EGFP pattern in EGFP KI HeLa cells in which Cas9 expression had been turned off. First, tagging of Lamin A/C protein with EGFP led to marking specifically of the nuclear membrane (Figure 4C), which matches the location of Lamin A/C in the nuclear lamina underlying the inner nuclear membrane.^35^ Also, staining of the cells for Lamin A/C showed perfect overlap between the EGFP and Lamin A/C signal (Figure 4C, merge). The cellular EGFP pattern was markedly different in *VIM*-targeted cells, showing a more diffuse distribution of the EGFP signal, matching its role as a component of the cytoskeleton (Figure 4D). Also, in this case, we observed an overlap between the EGFP signal and stained VIM (Figure 4D, merge). For a region of interest (ROI) focusing on the nuclear membrane, the Pearson correlation of the pixel intensity from EGFP and the antibody stain consolidated the overlap between the EGFP signal and the signal from the respective antibodies for LMNA (Figure 4E). To capture the full representation of the cytoskeleton, we calculated the Pearson correlation of the entire image for all VIM samples, and again the overlap between the EGFP signal and the VIM antibody was clear (Figure 4F). We also verified insertion of the EGFP tag by PCR and Sanger sequencing of the resulting amplicon in populations and isolated KI clones (Figure S11). These data suggested that the fluorescence tag had been inserted correctly in the two targeted loci.

To quantify the level of correct HDR-based KI based on tagged protein localization patterns, a high-throughput analysis of EGFP-tagged Lamin A/C expression was carried out using imaging flow cytometry. By including two individually validated *LMNA* KI HeLaCas9 clones (Figure S11) and HeLaCas9 clone#10 expressing EGFP as a control (Figure 4G), a selection of key phenotypic criteria was identified, allowing us to distinguish between a diffuse EGFP signal and the correctly localized EGFP signal generated from the EGFP-tagged LMNA protein using ImageStream (Figure 4H). By including validated *LMNA* KI clones, one with the EGFP tag incorporated into both alleles and one carrying the tag only in one *LMNA* allele (Figure 4I), we identified the SD of EGFP intensity within a nuclear confined mask as the best distinguishing phenotypic feature between bi-allelic and mono-allelic KI events. By applying the analysis template (Figure S12) to the *LMNA* KI samples from both HeLaCas9 clones (Figure 4J), 92.6% of the cells from HeLaCas9 clone#10 were found to contain a correct KI event, whereas 83.5% of the cells in HeLaCas9#15 harbored a targeted insertion. With our analysis template, gating based on the SD within the EGFP channel promptly indicated the composition of EGFP tag at the *LMNA* loci of each cell, allowing cells with targeted insertion of the tag sequence in one locus to be distinguished from cells with the tag inserted in both *LMNA* loci (Figure 4K). Quantification of these gates defined 33% of the cells in HeLaCas9#10 as homozygous for the correct insert, whereas as many as 40% of EGFP-positive clone #15 cells harbored the insert in both targeted alleles (Figure 4L). Based on these analyses, we conclude that cells with stable Cas9 expression support potent EGFP KI after IDLV-directed delivery of the sgRNA, donor sequence, and hyPB^Exc+^ protein, resulting in bi-allelic EGFP gene tagging in up to 40% of treated cells from which the Cas9 cassette has been tracelessly removed.

## Discussion

In this study, we provide a novel approach for targeted gene insertion using CRISPR-Cas9-based HDR repair. By delivering an sgRNA expression cassette together with a donor template in IDLVs loaded with excision-only piggyBac transposase proteins, we exploit high levels of stable endogenous Cas9 expression and the capacity to switch off Cas9 expression by potent excision of the DNA transposon as the source of Cas9. By combining the advantages of a high steady-state level of Cas9 production with a transposase-based off switch, we facilitate CRISPR-Cas9-based HDR followed by switching off Cas9 expression, eventually leaving cells without traces of the editing procedure. Using this approach, levels of Cas9 are consistently high until the effects of transposon excision kick in. Because of the expected delay in production of Cas9/sgRNA complexes (because of reverse transcription of the lentiviral vector RNA), one may speculate that targeted DNA cutting by this approach is timed with availability of the reverse-transcribed donor, leading to efficient HDR. At least, our data suggest that simultaneous delivery of preformed Cas9/sgRNA RNP complexes with an IDLV donor (that needs to undergo reverse transcription) does not support the same level of KI and, under these conditions, strongly depend on inhibition of NHEJ to facilitate tag insertion by HDR.

Initially, we demonstrated the excision capacity and integration deficiency of the hyPB^Exc+^ transposase. In line with a previous report,^26^ the protein alterations resulted in increased excision rates from episomal and genomically integrated transposon donors compared with the unaltered transposase control. By fusing the hyPB^Exc+^ variant to the N terminus of the GagPol polypeptide, we showed efficient excision of genomic integrated transposon cassettes by lentiviral protein transduction, supporting our previous findings of high DNA transposon mobility in cells treated with hyPBase-loaded LVNPs.^31^ After LVNP-mediated administration of the hyPB^Exc+^ variant, transposition activity was reduced to background levels (Figure 1C), suggesting that only transposon excision, and not insertion, was supported by LVNP-delivered hyPB^Exc+^. We took advantage of the ability of IDLVs carrying GagPol polypeptides with a C-terminal fusion protein to co-package a transfer vector, initially to deliver an sgRNA expression cassette. By delivering such IDLVs carrying the hyPB^Exc+^ transposase and the sgRNA cassette to HeLa cells expressing Cas9 from an integrated piggyBac transposon context, we demonstrated highly efficient targeted gene knockout. After transduction with IDLVs loaded with the hyPB^Exc+^ variant, we were able to enrich for FIAU-resistant cells, indicating that the transposon cassette had been successfully mobilized from the genome without re-insertion. With effective delivery of sgRNAs and equally effective removal of the transposon, this approach provides a high level of CRISPR-Cas9-mediated KO with concomitant transposon excision and switching off of Cas9 production.

The capacity of cells to recover from DSBs in the genome is a well-described phenomenon. To maintain genomic stability, the NHEJ machinery has been shaped by evolution for fast DSB repair but at the cost of fidelity.^36^, ^37^, ^38^ With the development of CRISPR-Cas9, the ability to precisely introduce DSBs combined with quick but erroneous repair results in a highly efficient platform for generating targeted gene knockout. Aiming to utilize the CRISPR-Cas9 system for HDR, the alertness of the NHEJ system becomes a challenge, as evident from recent studies of the dynamics behind repair of Cas9-induced DSBs.^18^^,^^39^ Aware of the prevalence of NHEJ-directed DSB repair, we wanted to utilize IDLVs to develop a novel approach for achieving site-specific insertion of foreign DNA into the genome. In our setup, the timing of Cas9-induced cleavage and the availability of an HDR template were automatically synchronized by conversion of single-stranded vector RNA to DNA by reverse transcription, ensuring that Cas9 and sgRNA complexes can only form when reverse transcription has been completed. We hypothesized that formation of complexes consisting of endogenously expressed Cas9 and sgRNA produced upon reverse transcription would support timed availability of donor DNA for DNA repair and gene insertion by HDR upon targeted DNA cleavage. By simultaneously neutralizing the source of Cas9 (by hyPB^Exc+^-directed transposon excision), the window of Cas9 cleavage was restricted, allowing only short-term DNA cleavage activity. This “footprint”-free setup allows gene insertion or gene tagging to be established through a single treatment requiring only establishment of Cas9-expressing cell lines using the regular piggyBac transposon system. We explore the basics of this editing approach in commonly used HeLa cells, but the protocol is applicable to any cell type compatible with transfection and transduction. We envision that this system could be employed in more advanced cell systems, including iPSCs. The HeLa cell lines we established here can be used to efficiently tag any gene of interest in the genome. The only requirements are (1) that IDLVs are produced with packaging constructs allowing co-packaging of hyPB^Exc+^ protein and (2) that a vector encoding the desired sgRNA and containing the appropriate homology arms is produced. Adapting the approach for use in a cell line of interest involves engineering of Cas9-expressing cells. However, after this initial investment, our approach does not require AAV production, which is not established as a standard setup in many labs, or purchase of recombinant Cas9 or synthetic guide RNAs.

In the work presented here, we demonstrate highly precise KI by simultaneous delivery of the hyPB^Exc+^ transposase, the sgRNA expression cassette, and a donor template carrying the tag sequence between relevant homology arms. Hence, co-delivery of all three components creates the conditions for precise KI of the tag sequence in relevant endogenous loci. By selecting with FIAU, we enriched for cells devoid of the Cas9-encoding transposon, yielding edited cells without Cas9. We believe that this method can serve as a platform for generating a wide range of cell models with tailored KI of various genomic modifications, including tagging of proteins, locus-directed gene repair, and site-directed cDNA insertion. Considering the packaging capacity of lentiviruses (∼10 kb), this method is potentially suited for KI applications where larger HDR templates, including entire genes or tags, are desired. With few steps of preparation, the system allows users to conveniently target and tag any locus in the genome using an approach based only on a single step of IDLV transduction.

## Materials and methods

### Nomenclature of lentiviral vectors and protein-loaded lentiviral particles

IDLVs are lentiviral vectors containing D64V integrase and vector RNA. IDLVs loaded with foreign protein, such as hyPBase, but that also carry vector RNA (for example, containing the EGFP gene driven by the phosphoglycerate kinase (PGK) promoter) are referred to as IDLV-hyPBase/PGK-EGFP. Protein-loaded particles, which do not carry vector RNA, are referred to as LVNPs with potential indication of which protein they contain; e.g., LVNP-hyPBase.

### Plasmid cloning

The pCMV-hyPB^Exc+^ plasmid expressing the hyperactive, excision-only piggyBac variant, driven by the cytomegalovirus (CMV) promoter, was made by overlap extension PCRs to introduce the R372A, K375A, and D450A missense mutations. The resulting PCR product was cloned into EcoRI- and BsrGI-digested pCMV-hyPBase.^30^ pPBT/PGK-Puro has been described previously.^40^ The pT2/CMV-EGFP.PB/PTK reporter vector was created by first inserting an overlap extension PCR of a 3′EGFP.pA-RIR (right inverted repeat) fragment from pT2/CMV-EGFP (unpublished data) and a 3′ terminal repeat (TR) fragment from pMCS-AAT-PB:PGKPuroΔTK^25^ into NotI– and SfiI-digested pMCS-AAT-PB:PGKPuroΔTK. The resulting vector was subsequently digested with SgsI and NsiI, and a left inverted repeat (LIR)-CMV-5′EGFP fragment was inserted to create pT2/CMV-EGFP.PB/PTK. pInt^−^PCS-hyPB^Exc+^ was constructed by insertion of a DraIII-digested hyPB^Exc+^ fragment from pCMV-hyPB^Exc+^ into pInt^−^PCS-hyPBase.^31^ pPBT/EFS-Cas9-PuroTK was cloned by insertion of a PCR-amplified PuroTK fragment from pCCL/pBT.PuroTK-CMV (unpublished data) into BamHI- and NdeI-digested pPBT/EFS-Cas9-Blast (unpublished data) using NEBuilder HIFI DNA Assembly (New England Biolabs, Ipswich, MA, USA). pLV/Guide-mCherry was cloned by digesting pLV/Guide-Puro^41^ with BsiWI and Mlul and amplifying mCherry from pT2/CMV-SerpinG1-linker-mCherry. Similarly, plenti/Cas9-mCherry was cloned by digesting plentiCas9-Blast^42^ with BamHI and EcoRI and inserting the mCherry sequence amplified from pLV/Guide-mCherry by a nested extension PCR to include P2A between Cas9 and mCherry. The resulting fragments were assembled using NEBuilder HIFI DNA Assembly (New England Biolabs). The lentiviral donor vectors for HDR were generated by first replacing the EFS-mCherry expression cassette of pGuide-mCherry with an EGFP PCR fragment to create pLV/EGFP-U6-sgRNA. This was done by PCR amplification of EGFP from pCCL/PGK-EGFP and insertion into Cfr9I -and MluI-digested pGuide-mCherry by NEBuilder HiFi DNA Assembly (New England Biolabs) using the manufacturer’s instructions. LHA and RHA were subsequently synthesized as GenParts DNA fragments (GenScript) and inserted into BstXI- or BshTI-digested pLV/EGFP-U6-sgRNA by NEBuilder assembly for LHA and RHA insertion, respectively. The AAV donor vectors for HDR were generated by digesting pAAV-MCS-BGHpA with NotI and inserting LHA-EGFP-linker-RHA, PCR-amplified from the relevant lentiviral vectors, for LMNA and VIM, respectively. For pAAV-‘5GFP-KI, only the EGFP-linker was amplified. The PCR fragments and digested vector were assembled by NEBuilder HIFI DNA Assembly.

### Cell culture work

HEK293, HEK293T, and HeLa cells were cultured under standard conditions at 37°C in 5% CO_2_ in Dulbecco’s modified Eagle’s medium (DMEM) (Sigma-Aldrich, St. Louis, MO, USA) supplemented with 5% fetal calf serum, penicillin (100 U/mL), and streptomycin (100 μg/mL).

### Generation of DNA transposon-containing cell lines

For generation of HEK293 and HeLa reporter cell lines harboring the T2/CMV-EGFP.PB/PTK transposon cassette, 2.5 × 10^5^ cells/well were seeded 1 day prior to transfection in 6-well plates. Cells were co-transfected with 450 ng pT2/CMV-EGFP.PB/PTK and 50 ng of pCMV-SB100X using X-tremeGENE 9 (Roche, Basel, Switzerland) or TurboFect (Thermo Fisher Scientific, Waltham, MA, USA) for transfection of HEK293 or HeLa cells, respectively, according to the manufacturer’s instructions. One day after transfection, the cells were split into P10 dishes with appropriate dilutions, and the following day, the medium was changed to medium supplemented with 1 μg/mL puromycin (Sigma-Aldrich). Single puromycin-resistant colonies were isolated and expanded for further experiments. For generation of HeLa PB/Cas9-PuroTK cell lines, HeLa cells were transfected with 900 ng pPBT/EFS-Cas9-PuroTK and 100 ng pCMV-hyBPase using TurboFect, and single clones were isolated as described above.

### Production of IDLVs and LVNPs

Production of protein-transducing LVNPs (devoid of vector RNA) was carried out by standard calcium phosphate transfection of lentiviral packaging plasmids into HEK293T cells seeded the day before in 15-cm dishes at 1 × 10^7^ cells per dish. Co-transfections were carried out using 7.26 μg pRSV-Rev, 9.07 μg pMD2.G, and 62.92 μg GagPol-encoding plasmid. Standard IDLVs or IDLVs containing a foreign protein (also carrying vector RNA) were generated in a similar manner but with the following amounts of plasmids: pRSV-Rev, 7.26 μg; pMD2.G, 9.07 μg; GagPol-encoding plasmid, 31.46 μg; and lentiviral transfer vector, 31.46 μg. One day after transfection, the medium was replaced, and 2 days after transfection, the supernatant was harvested by filtration through a 0.45-μm filter (Sarstedt, Nümbrecht, Germany). Viral particles were pelleted by ultracentrifugation of viral supernatant through a 4-mL 20% sucrose cushion at 25.000 rpm at 4°C for 2 h, followed by resuspension of the pelleted virus in Dulbecco’s PBS (DPBS)without calcium and magnesium. The yield of each vector preparation was determined by p24 ELISA using kits provided by Zeptometrix (Buffalo, NY, USA) or XpressBio (Thurmont, MD, USA), following the manufacturers’ protocols.

### Production of AAV and titer determination

For each AAV preparation, 11 × 10^6^ HEK293T cells were seeded in each of 10 15-cm dishes 24 h prior to transfection. Before transfection, the medium was changed to 20 mL fresh pre-warmed medium supplemented with 1 mM sodium butyrate. Transfections were carried out in OptiMEM using PEI and 22 μg pDGM6 and 6 μg vector plasmid for each 15-cm dish. After 72 h, the cells were harvested using a cell scraper and pelleted at 1,258 × *g* for 15 min at 4°C. Cell pellets containing all cells from 10 dishes were resuspended in 5 mL lysis buffer (10 mM Tris and 2 mM MgCl [pH 8]). The lysed pellets underwent three freeze/thaw cycles and were subsequently treated with 200 U/mL Turbonuclease (Sigma-Aldrich, T4330-50KU) at 37°C for 45 min. The AAV vectors were purified using a standard iodixanol gradient in 13.5-mL quick-seal ultracentrifuge tubes (Beckman Coulter, 342413), and centrifuged in a Beckman Type 70.1Ti rotor (Beckman Coulter, 342184) at 48,000 rpm for 2 h at 18°C using a Beckman Coulter L8-70M ultracentrifuge. After centrifugation, approximately 1 mL was extracted between intersection of the 40% and 58% layer using a 20G needle and transferred to a tube containing 14 mL PBS with 5% sorbitol and 0.001% Pluronic. The resulting 15-mL AAV-containing solution was concentrated using an Amicon Ultra-15 centrifugal filter unit (Sigma-Aldrich, UFC910096) by centrifuging the solution through the filter at 3,000 × *g*, until around 200–300 μL was retained in the filter.

### Colony formation assays

Quantification of transposition activity by colony formation assays has been described previously.^31^ Briefly, HeLa cells were seeded at a 2.5 × 10^5^ cells/well in 6-well plates and, on the following day, transfected with 900 ng pPBT/PGK-Puro transposon and 100 ng of pCMV-hyPBase, pCMV-hyPB^Exc+^, or pCMV-hyPBmut using TurboFect (Sigma-Aldrich). For the colony formation assay using transposase-loaded LVNPs, cells were first transfected with 900 ng pPBT/PGK-Puro and transduced 24 h after transfection with 1 μg p24 of LVNP-hyPBase, LVNP-hyPB^Exc+^, or LVNP-hyPBmut in the presence of Polybrene (8 μg/mL). The day after transfection/transduction, the cells were split into P10 dishes, and the medium was supplemented with 1 μg/mL puromycin. For the excision assays, cells were transduced with LVNP-hyPB^Exc+^, IDLV-hyPB^Exc+^/sgRNA-AFF1, or LVNP-hyPBmut corresponding to 1 μg p24 and split into P10 dishes. One week after transduction, the medium was changed to medium supplemented with 1.6 μM FIAU. Two to three weeks later, puromycin- and FIAU-resistant colonies were large enough to be visualized by staining with 0.6% methylene blue (Sigma-Aldrich) and counted.

### Evaluation of transposon excision by flow cytometry

For testing transposon excision from a transiently transfected reporter, HEK293 cells were seeded in 6-well plates at a density of 2.5 × 10^5^ cells/well 1 day before transfection. For each transfection, 1.5 μg pT2/CMV-EGFP.PB/PTK was co-transfected with 1.5 μg of pCMV-hyBPase, pCMV-hyPB^Exc+^, or pCMV-hyPBmut using X-tremeGENE 9 (Roche) according to the manufacturer’s protocol. For testing genomic excision in HeLa and HEK293 reporter cell lines, the cells were seeded as above and, the next day, transfected with 1 μg pCMV-hyPB^Exc+^ or transduced with 1 μg p24 of LVNP-hyPBase or LVNP-hyPB^Exc+^. For the repeated dosing experiment, the cells were transduced for up to consecutive days with 1 μg pCMV-hyPB^Exc+^ or LVNP-hyPBmut. Four days after transposase delivery, EGFP expression was analyzed. The cells were trypsinized, washed in DPBS, fixed in 2.5% formaldehyde, and resuspended in 250 μL DPBS for analysis by flow cytometry. Flow cytometry analysis was carried out at the FACS Core facility of Aarhus University on a BD LSRFortessa (Becton Dickinson, Franklin Lakes, NJ, USA) equipped with 4 lasers and 16 detectors and a BD High-Throughput Sampler or a Novocyte 2100 analyzer (Agilent Technologies, Santa Clara, CA, USA). All flow data were analyzed using FlowJo v.10.0.7.

### Southern blotting

DNA was extracted using a standard NaCl/EtOH precipitation protocol. For each clone, 15 μg of gDNA was digested overnight. T2/CMV-EGFP.PB/PTK clones were digested with NsiI and ScaI, and pPBT/EFS-Cas9-PuroTK clones were digested with KpnI and DraI before gel electrophoresis and vacuum blotting. For T2/CMV-EGFP.PB/PTK clones, a probed targeting the puromycin resistance gene was prepared by BsiwI and PshAI digestion of T2/CMV-EGFP.PB/PTK, resulting in a 1,462-bp probe. For pPBT/EFS-Cas9-PuroTK clones, BstXI and Eco32I were used for digestion of pPBT/EFS-Cas9-PuroTK, resulting in a 1,243-bp fragment from the puromycin resistance gene. Both probe fragments were randomly labeled using the Prime-It random primer labeling kit (Agilent Technologies) according to the manufacturer’s instructions using α-^32^P dCTP (PerkinElmer).

### ddPCR

For estimation of donor copy numbers and copy numbers of integrated transposon cassettes in recipient cells, DNA was extracted using a standard NaCl/EtOH precipitation protocol. For each sample 1 μg of genomic DNA was digested overnight with HindIII in 20 μL total. Samples were diluted 10-fold, and 5 μL was used as a template with 2× ddPCR SuperMix for Probes (No dUTP) (Bio-Rad, Hercules, CA, USA; 1863023). Donor copy numbers were quantified with primers and probes targeting GFP (HEX) and albumin (FAM) (TAG Copenhagen). The number of integrated PBT/EFS-Cas9-PuroTK transposons was determined using primers and probes targeting Puro (FAM) and albumin (HEX) (TAG Copenhagen). Sequences are available in Table S1. The PCR was set up according to the manufacturer’s instructions with an annealing temperature of 60°C.

The titer of the AAV preparations was determined using a primer/probe set with FAM (Integrated DNA Technologies) targeting the AAV2 inverted terminal repeat (ITR) described by Wang et al.^36^ 5 μL concentrated AAV was treated with 10 U DNAseI (Thermo Fischer Scientific, EN0521) in a total reaction volume of 96 μL at 37°C for 30 min. Then 4 μL of 0.5 M EDTA (Thermo Fisher Scientific) was added, followed by 50 μL QuickExtract (Lucigene, QE09050). The solution was incubated for 6 min at 60°C, followed by 10 min of incubation at 100°C. For each preparation, 1 × 10^−4^, 5 × 10^−4^, and 1 × 10^−5^ dilutions of the lysed AAV were used. 5 μL diluted AAV was used as a template in the ddPCR reaction, as described above.

For AAV titer and donor copy number assessments, droplets were made with Droplet Generation Oil for Probes (Bio-Rad, 1863005) and a QX200 droplet generator (Bio-Rad). The droplets were loaded into a ddPCR 96-well plate (Bio-Rad, 12001925) and sealed with PCR plate heat seal foil (Bio-Rad, 1814040) using a PX1 PCR plate sealer (Bio-Rad). The plates were read using a QX200 droplet reader (Bio-Rad).

GFP copy number determination and AAV titer were determined with QuantaSoft Analysis Pro. The concentration of virus genomes in the undiluted solution was then calculated. The final titer was calculated as the average concentration of virus genomes across all of dilutions. GFP donor copy number per cell was calculated assuming an albumin copy number of two per cell.

### Analysis of indel rates

Genomic DNA was extracted using a standard NaCL/EtOH precipitation protocol. The targeted region of *AFF1* was PCR amplified, and amplicons were purified on a 1% agarose gel. The PCR products were then Sanger sequenced (Eurofins Genomics) and analyzed using the ICE software (ice.synthego.com).

### Cas9/sgRNA RNP nucleofection

Cas9/sgRNA RNP complexes were generated by mixing 6 μg of recombinant spCas9 (Alt-R spCas9 nuclease V3; Integrated DNA Technologies, NJ, USA) with 3.2 μg of synthetic sgRNAs (Synthego, CA, USA) in 2 μL total and incubated for 15 min at 25°C. RNP complexes were added to 3 × 10^5^ cells in 18 μL OptiMEM, and the resulting 20-μL reactions were electroporated on a Lonza 4D-Nucleofector with P3 solution settings and pulse code CN 115.

### EGFP KI

HeLa PB/Cas9-PuroTK clones were seeded into 24-well plates at 2 × 10^4^ cells/well and transduced with hyPB^Exc+^-IDLV/donor vectors. One week after transduction, the medium was supplemented with 1.6 μM FIAU. Resistant cells were expanded and analyzed by flow cytometry as described above. Experiments performed in HeLa PB/Cas9-PuroTK cells with IDLV/donor vectors were not exposed to FIAU. For KI by plasmid co-transfection, cells were seeded at 5 × 10^4^ cells/well and transfected using Turbofect. A total of 1 μg plasmid DNA was used, with 900 ng donor/sgRNA vector and 100 μg plentiCas9-mCherry vector. Cas9/sgRNA RNP-based EGFP KI was carried out by nucleofection and subsequent seeding of 2 × 10^4^ cells/well in 24-well plates. Immediately after nucleofection, cells were transduced with IDLV/donor vectors (40 ng, p24) or AAV6 serotype AAV/donor vectors at an MOI of 1 × 10^5^, as reported previously as optimal conditions for HeLa cells.^43^ After nucleofection and transduction, cells were kept in 250 μL total for the first 24 h. In experiments with M3814 (Chemietek, IN, USA; CT-M3814), 2 μM was used.

### Confocal microscopy

After transduction with IDLV-hyPB^Exc+^/EGFP-5′LMNA KI or IDLV-hyPB^Exc+^/EGFP-5′VIM-KI and FIAU selection, cells were seeded on collagen-coated (Sigma-Aldrich) coverslips (VWR, 631-0125) in a 6-well plate at a density of 1.5 × 10^5^ cells/well 1–2 days prior to fixation. The cells were fixed for 10 min in 4% paraformaldehyde (Thermo Fisher Scientific) and washed three times for 3 min each time in DPBS. The LMNA KI HeLa cells were then kept in ice-cold 70% ethanol in the freezer and washed three times for 3 min each time in DPBS prior to permeabilization in 0.2% Triton X-100 (Sigma-Aldrich) in 1× PBS for 12 min. The cells were then blocked for 25 min in 2% BSA in DPBS and washed twice with 0.05% Triton X-100 in DPBS before the cells were stained with anti-Lamin monoclonal primary antibody (Santa Cruz Biotechnology, sc-376248) diluted 1:50 in DPBS supplemented with 2% BSA and 0.05% Triton X-100. This was followed by three 5-min washes in 0.05% Triton X-100 in DPBS prior to labeling of the primary Lamin antibody with Alexa Fluor-647-conjugated secondary antibody (Thermo Fisher Scientific) diluted 1:400 in DPBS supplemented with 2% BSA and 0.05% Triton X-100. After staining with the secondary antibody, the cells were washed three times for 5 min each time with 0.05% Triton X-100 in DPBS. After fixation in 4% paraformaldehyde (PFA), VIM KI HeLa cells were permeabilized in 0.5% in Triton X-100 (Sigma-Aldrich) in 1× DPBS for 10 min and then blocked in 2% BSA in DPBS for 1 h. The cells were then washed three times for 1 min each time in DPBS before staining with anti-VIM antibody (Abcam, ab20346) diluted 1:100 in DPBS supplemented with 1% BSA. Before and after labeling of the primary VIM antibody with Alexa Fluor 647-conjugated secondary antibody (Thermo Fisher Scientific) diluted 1:400 in DPBS supplemented with 1% BSA, the cells were washed three times for 1 min each time in DPBS. After staining, the cells were washed in ddH_2_O and stained with 2 μg/mL Hoechst, for visualization of nucleic acids. After staining, the coverslips were washed in ddH_2_O and mounted on microscope slides with Glycergel mounting medium (Agilent Technologies; Dako, Glostrup, Denmark). Images were acquired with a Carl Zeiss LSM800 inverted confocal microscope equipped with a sensitive GaAsP detector, three detectors, one Airyscan detector, one transmitted detector, and a Plan-Apochromat 63×/1.40 oil differential interference contrast (DIC) M27 objective. Airyscan imaging processing and Pearson correlation analysis were performed using the Carl Zeiss Zen Desk software.

### Western blot analysis

1 × 10^6^ cells were lysed in RIPA buffer supplemented with 10 mM NaF and 1× complete protease inhibitor cocktail (Roche) and incubated for 15 min on ice prior to sonication of the lysate with six pulses of 30 s. The lysate was centrifuged at 4°C for 15 min at 13,000 rpm, and the supernatant was moved to a new tube. The protein lysate was denatured in XT Sample Buffer supplemented with XT Reducing Agent (Bio-Rad), separated by SDS-PAGE, and blotted onto a polyvinylidene fluoride membrane. The membrane was blocked in 5% skim milk (Sigma) in Tris-buffered saline (TBS)/0.05% Tween 20 for 1 h, followed by overnight incubation with FLAG primary antibody (Sigma-Aldrich). The blots were washed and incubated with anti-mouse secondary antibody (Dako) and visualized by chemiluminescence using Clarity Western enhanced chemiluminescence (ECL) substrate (Bio-Rad). The membrane was washed with stripping buffer (Thermo Fisher Scientific), and the membrane was incubated overnight with anti-β-actin antibody (Abcam), followed by anti-mouse secondary antibody and visualization.

### Imaging flow cytometry

Cells were seeded at 1.8 × 10^6^/p10 dish 2 days prior to preparation for ImageStream analysis. Cells were washed in DPBS−/− and trypsinized. After pelleting, cells were washed twice in DPBS−/− and fixed for 15 min at room temperature in 4% PFA. Finally, cells were stained with 0.2 μg/mL DAPI in DPBS−/− and resuspended in DPBS−/− and 1% BSA. Cells were run on an Amnis ImageStream Mark II (Luminex). Raw image data files were acquired at a 60× magnification in INSPIRE software (v.200.1.620.0, Luminex; laser power: 110 mW, 488-nm laser and 120 mW, 405-nm laser). Bright-field images were collected in channel 1 (Ch1) and Ch9, EGFP in Ch2, and DAPI in Ch7. Data were analyzed in IDEAS 6.3 (Luminex). After initial gating on healthy single cells in focus, the wizard Finding Best Feature was used to discriminate between cells displaying a negative or diffuse EGFP morphology and the correct LMNA EGFP-tagged morphology. We used the wizard Finding Best Feature to discriminate between cells with homozygous LMNA EGFP-tagged alleles and heterozygous LMNA EGFP-tagged alleles.

### Statistical analysis

Experimental data were analyzed using GraphPad Prism 9.4.0. When applicable, data were analyzed using unpaired t tests.

## Data availability statement

Data and original material presented in the study are available upon request from the corresponding author.

## Supplemental information

Document S1. Figures S1–S12 and Table S1

Document S2. Article plus supplemental information
