# Supplementary material for: CRISPR-Cas9-directed gene tagging using a single integrase-defective lentiviral vector carrying a transposase-based Cas9 off switch
Source: Mol Ther Nucleic Acids. 2022 Aug 4;29:563–76. doi: 10.1016/j.omtn.2022.08.005 (PMC9403905; doi:10.1016/j.omtn.2022.08.005)
Supplement: Document S1. Figures S1–S12 and Table S1 [file mmc1.pdf]

OMTN, Volume *29*

Supplemental information

CRISPR-Cas9-directed gene tagging using a single integrase-defective lentiviral vector carrying a transposase-based Cas9 off switch

Emil Aagaard Thomsen, Kristian Alsbjerg Skipper, Sofie Andersen, Didde Haslund, Thomas Wisbech Skov, and Jacob Giehm Mikkelsen

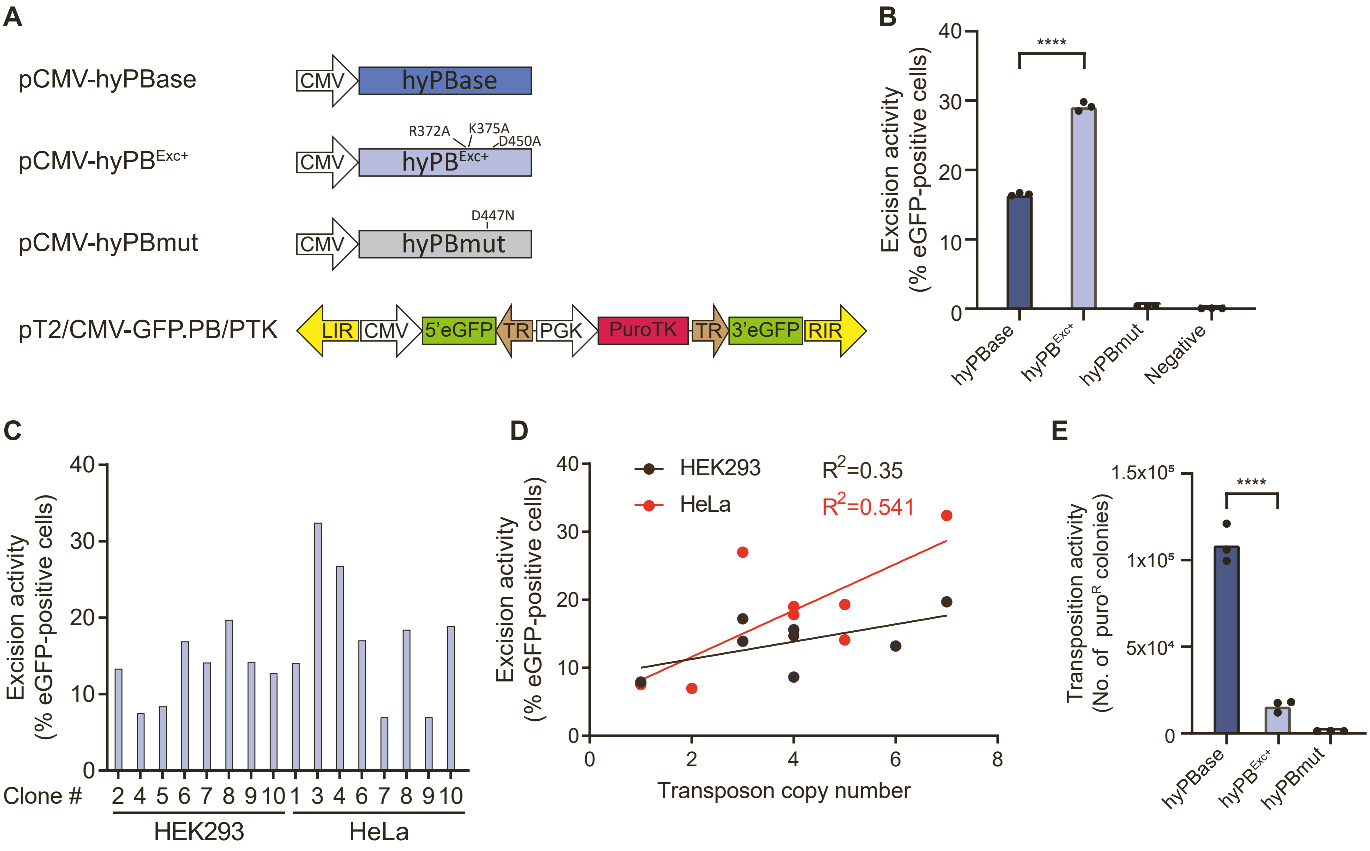

Figure S1. Development and evaluation of an excision-competent and integration-deficient hyPBase variant. (A) Schematic representation of the constructs used in the first part of this study. The sleeping beauty cassette is flanked by LIR and RIR (yellow), whereas the piggyBac cassette is flanked by TR (brown). **(B)** HyPB^{Exc+} markedly increases excision rates of episomal DNA. HEK293 cells were cotransfected with the eGFP excision reporter pT2/CMV-eGFP.PBT/PTK and plasmid DNA expressing either hyPBase, hyPB^{Exc+} or hyPBmut and subsequently analysed by flow cytometry, ****P<0.0001 (unpaired t-test). **(C)** Evaluation of single, isolated HEK293 and HeLa cell lines carrying the eGFP piggyBac excision reporter. Clones were transfected with the pCMV-hyPB^{Exc+}, and excision rates were estimated by flow cytometry. **(D)** Transposon excision efficiency only shows weak correlation with integrated transposon copy number. Transposon copy number estimated by Southern blotting and plotted against data shown in (C). Regression lines are shown as are the R^2 values for both HEK293 (blue) and HeLa (red) clones. **(E)** Comparison of PB transposition efficiency of hyPB protein variants. HeLa cells transfected with a pPBT/PGK-Puro transposon donor were transfected with plasmids encoding either hyPBase, hyPBmut or hyPB^{Exc+} protein and transposition was quantified by colony formation, ****P<0.0001 (unpaired t-test). Experiments were performed in biological triplicates (individual wells), bars represent mean with dots corresponding to individual replicates.

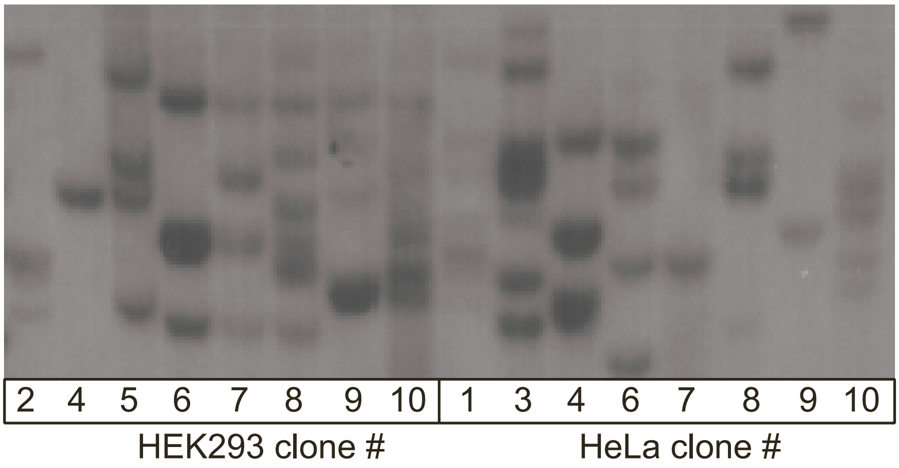

Figure S2. Copy number estimation in eGFP excision reporter cell lines by southern blotting. Detection of the Sleeping Beauty cassette T2/CMV-eGFP.PB/PTK by Southern blot for 8 different clones of each cell line.

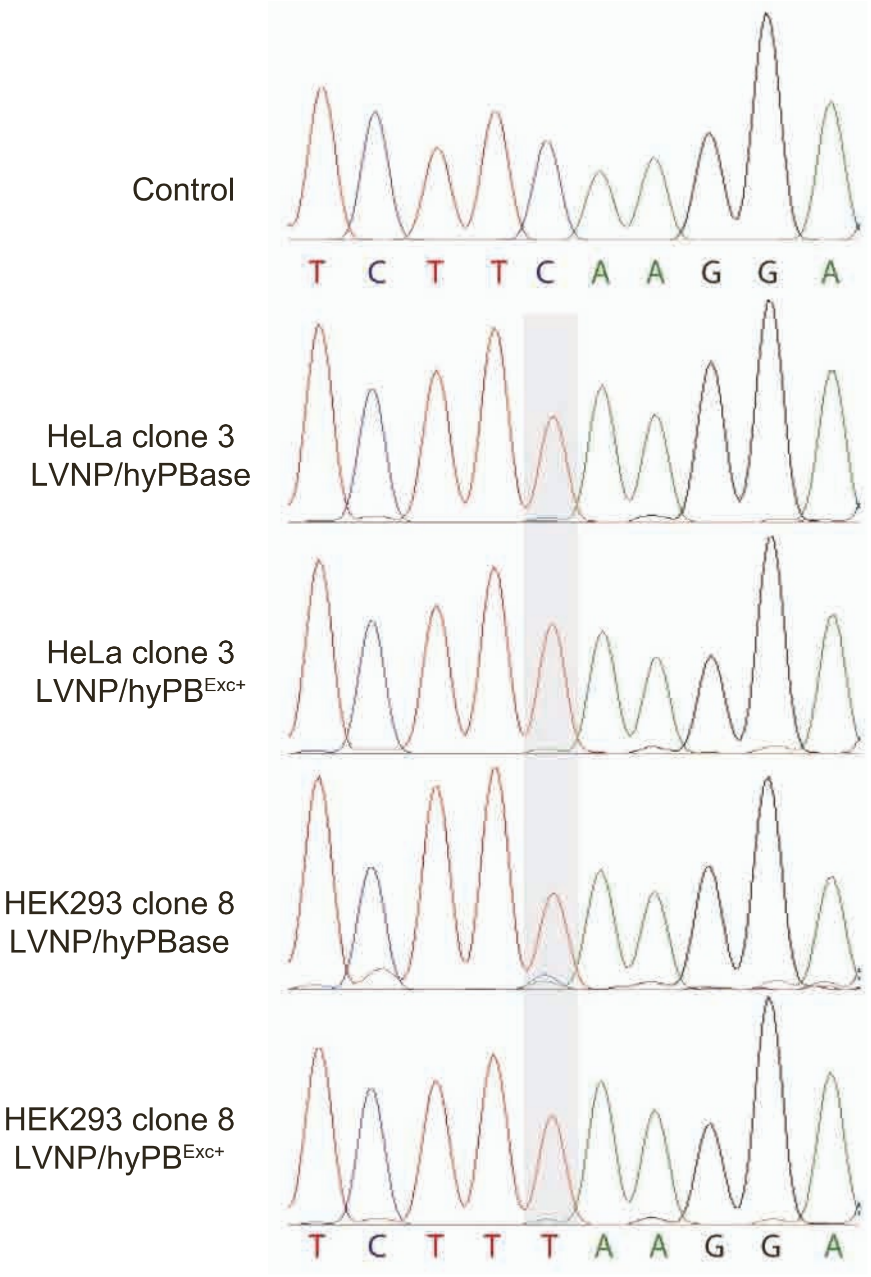

Figure S3. LVNP-delivered hyPB^{Exc+} mediates seamless genomic excision. Representative Sanger sequencing on genomic DNA from cells treated with hyPBase -or hyPB^{Exc+}-loaded LVNPs.

A

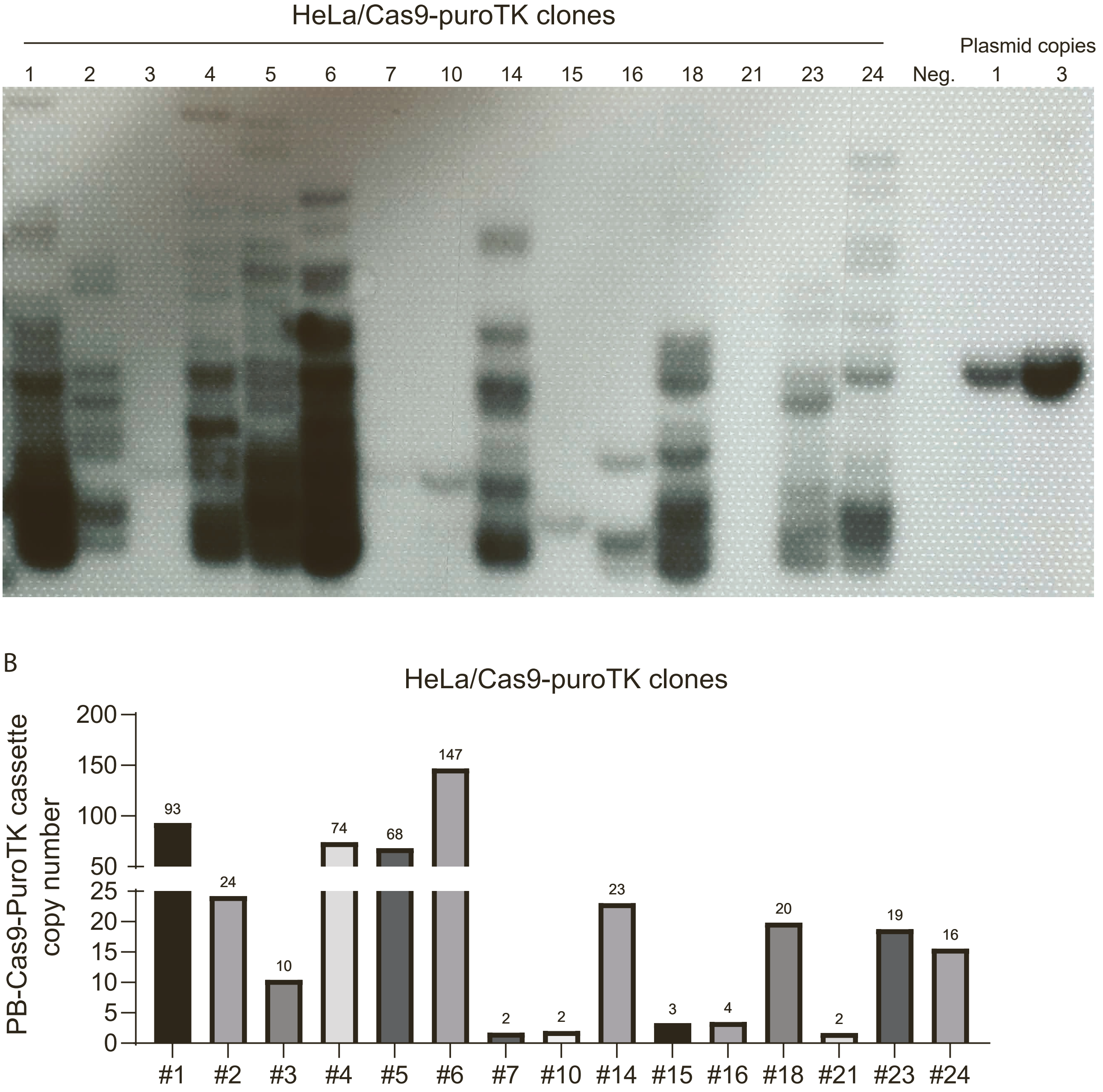

Figure S4. Copy number estimation of the PBT/EFS-SpCas9-puroTK cassette in HeLa cells. (A) Detection of the PBT/EFS-SpCas9-puroTK cassette by Southern blot in 15 different HeLa clones. (B) ddPCR-based quantification of the PBT/EFS-SpCas9-puroTK cassette in the 15 HeLa clones.

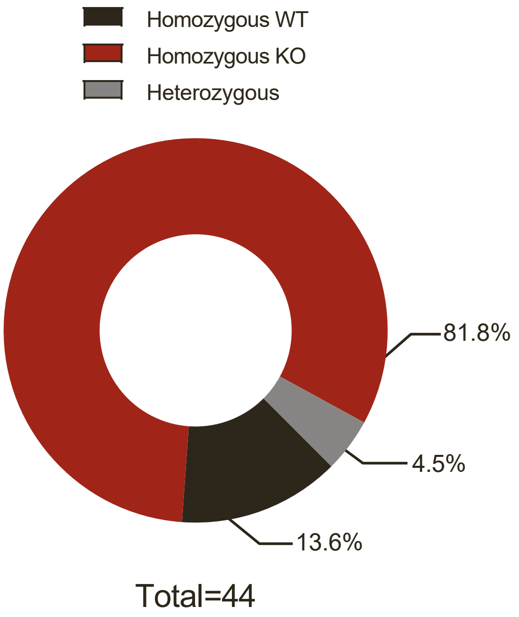

Figure S5. ***AFF1*** **genotype status in single clones.** Quantification of AFF1 genotype across 44 single clones derived from HeLa/Cas9#3 transduced with IDLV-hyPB^{Exc+}/ sgRNA.AFF1.

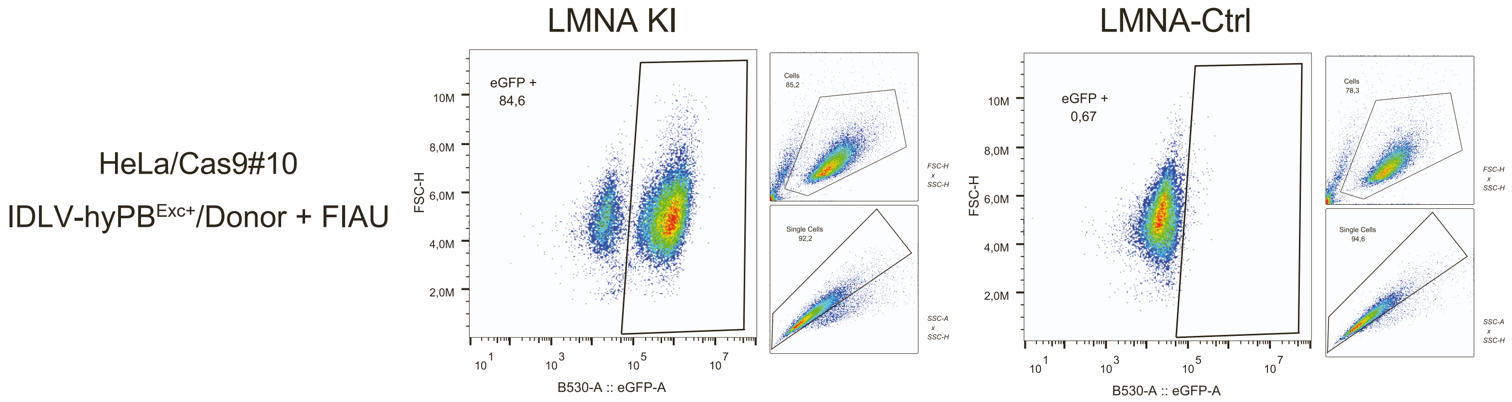

Figure S6. Flow cytometric quantification of eGFP-signal. Representative gating strategy used in the analysis of flow cytometric quantification of the eGFP positive fractions.

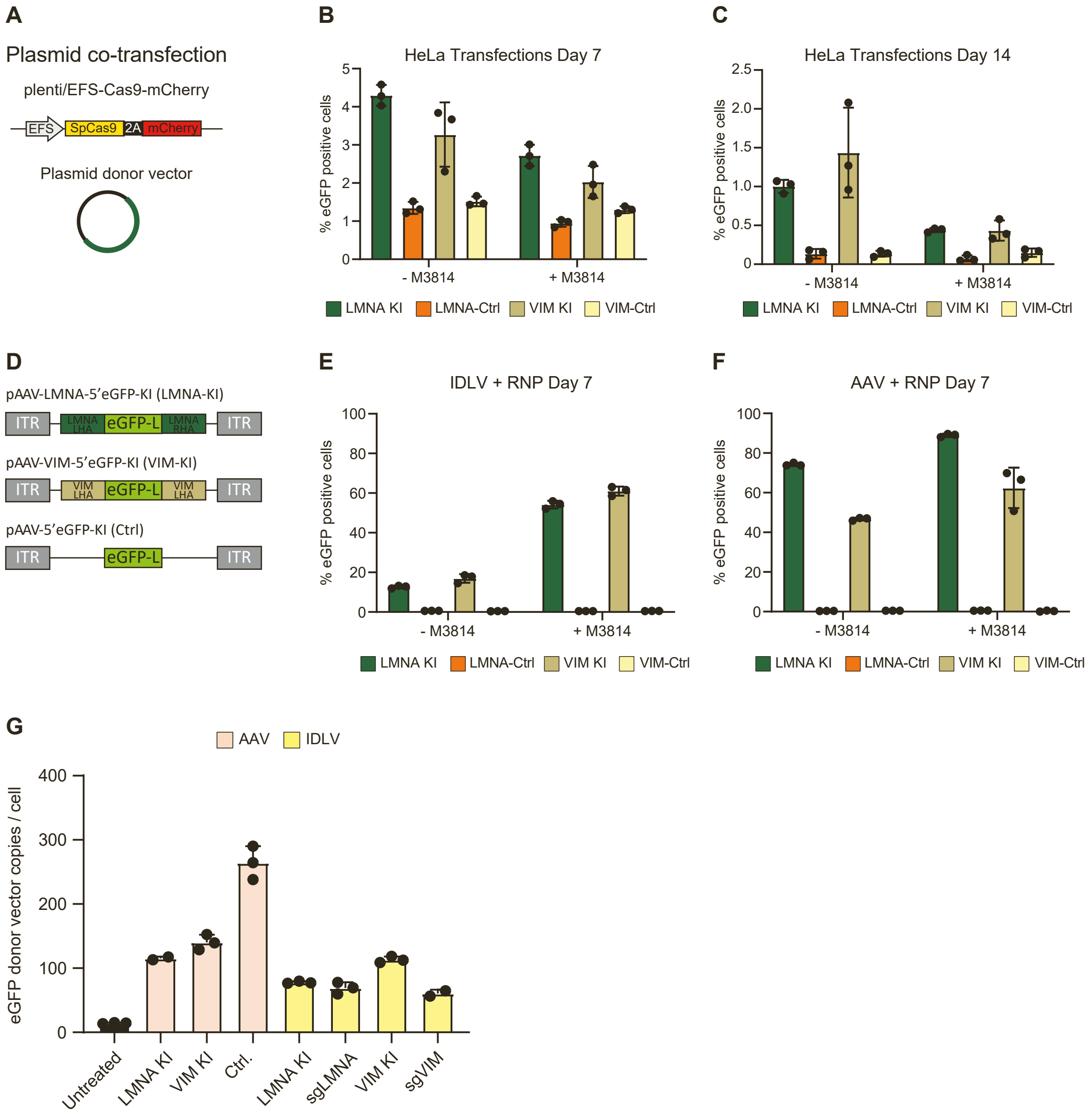

Figure S7. Cas9-mediated HDR-based eGFP-tagging of endogenous proteins. (**A**) Schematic presentation of the and cas9 expression donor vector for plasmid co-transfections. (**B-C**) HDR-based eGFP insertion into the *LMNA* locus or *VIM* locus by plasmid co-transfection in HeLa Cells, 7 days after transfection (**B**) and 14 days (**C**). (**D**) Vector schematics of AAV knock-in donor vectors with homology arms (KI) or without arms (Ctrl). (**E**) HDR-based eGFP insertion into the *LMNA* locus or *VIM* locus 7 days after IDLV donor delivery (40 ng P24) followed immediately by nucleofected with Cas9/sgRNA. (**F**) HDR-based eGFP insertion into the *LMNA* locus or *VIM* locus 7 days after AAV donor delivery (MOI of 1 x 10^5) followed immediately by nucleofected with Cas9/sgRNA. (**G**) Quantification of donor DNA copy numbers by ddPCR, 3 days after transduction with either IDLV or AAV donor.

A

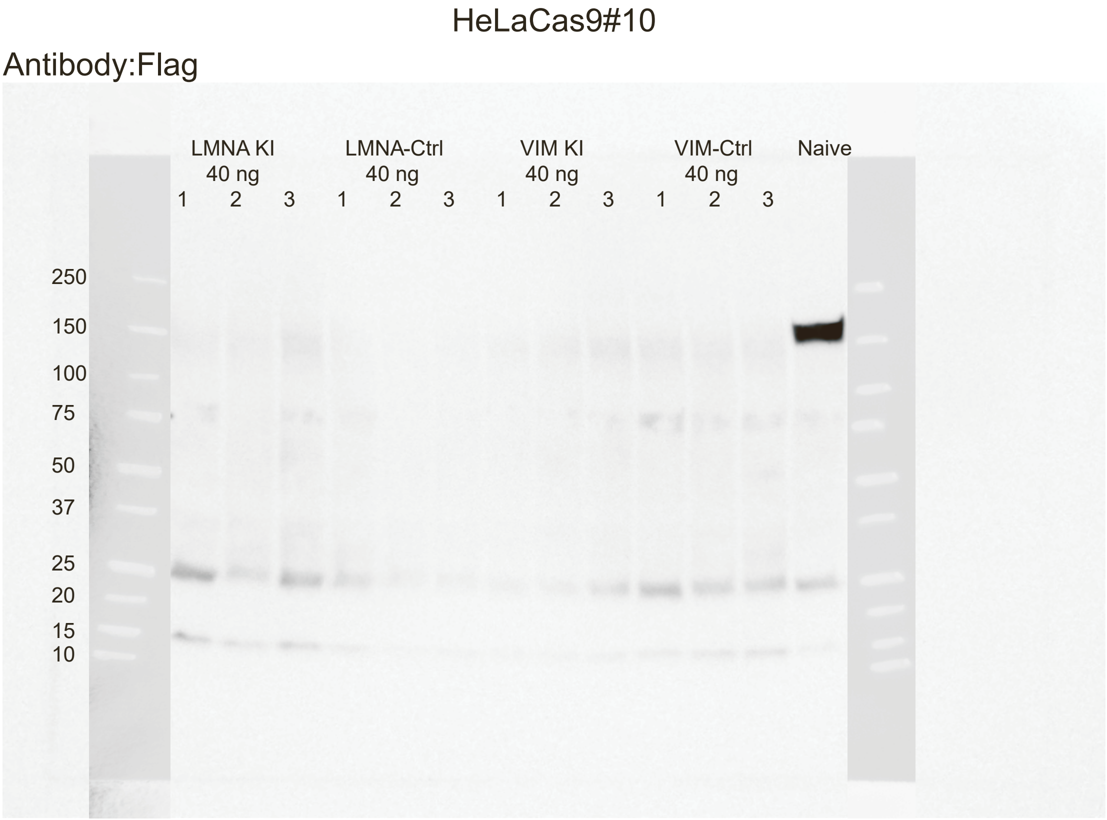

B

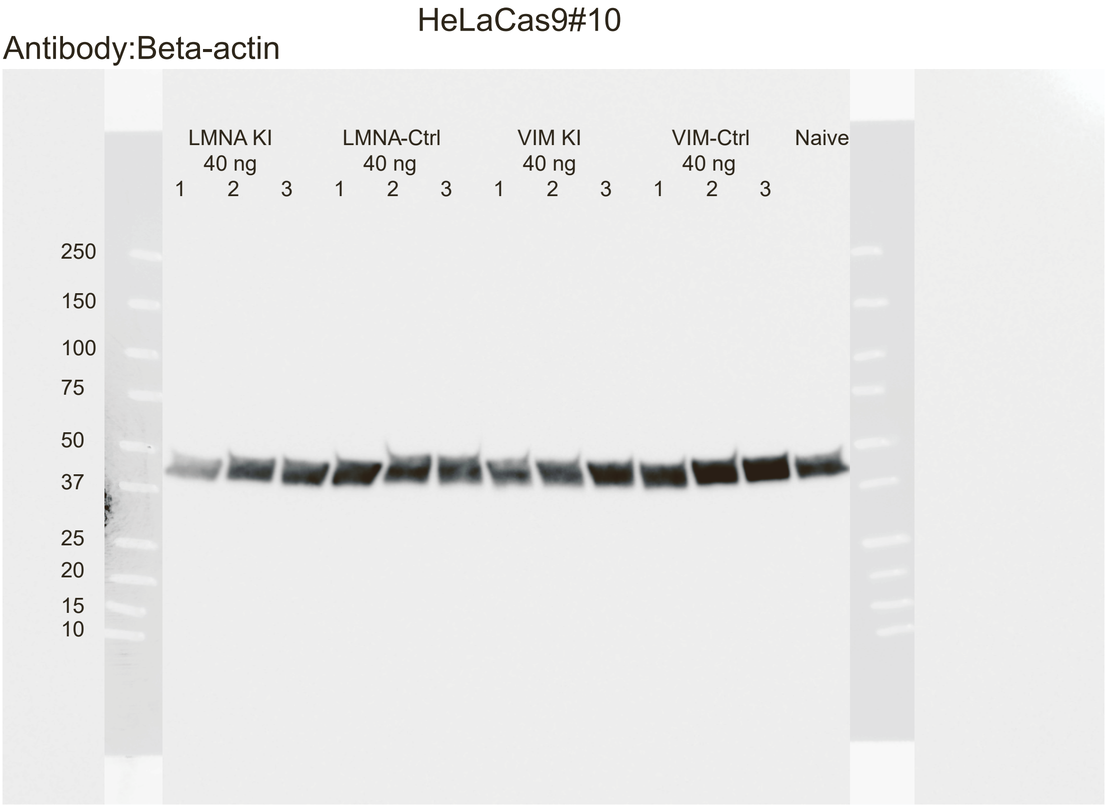

Figure S8. Uncropped western blot membranes from HeLaCas9#10. (A) Detection of Cas9 by primary antibody targeting the Flag-tag, fused to Cas9. **(B)** Detection of beta-actin, serving as a loading control.

A

HeLaCas9#15

Antibody:Flag

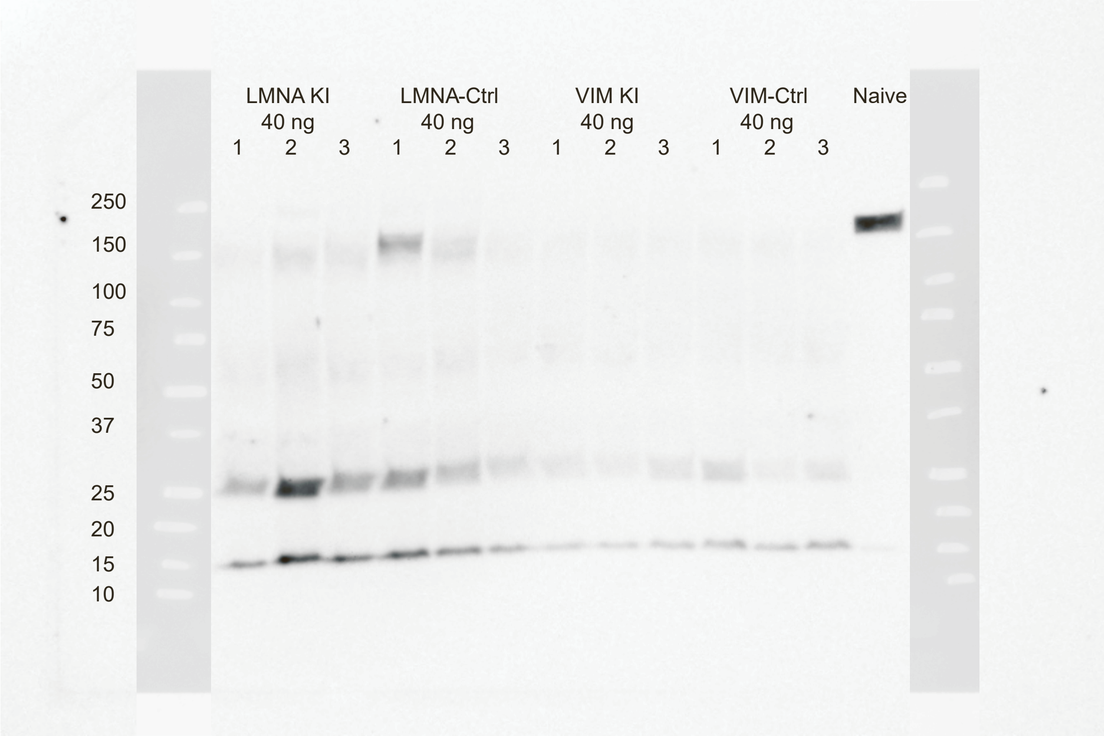

B

HeLaCas9#15

Antibody:Beta-actin

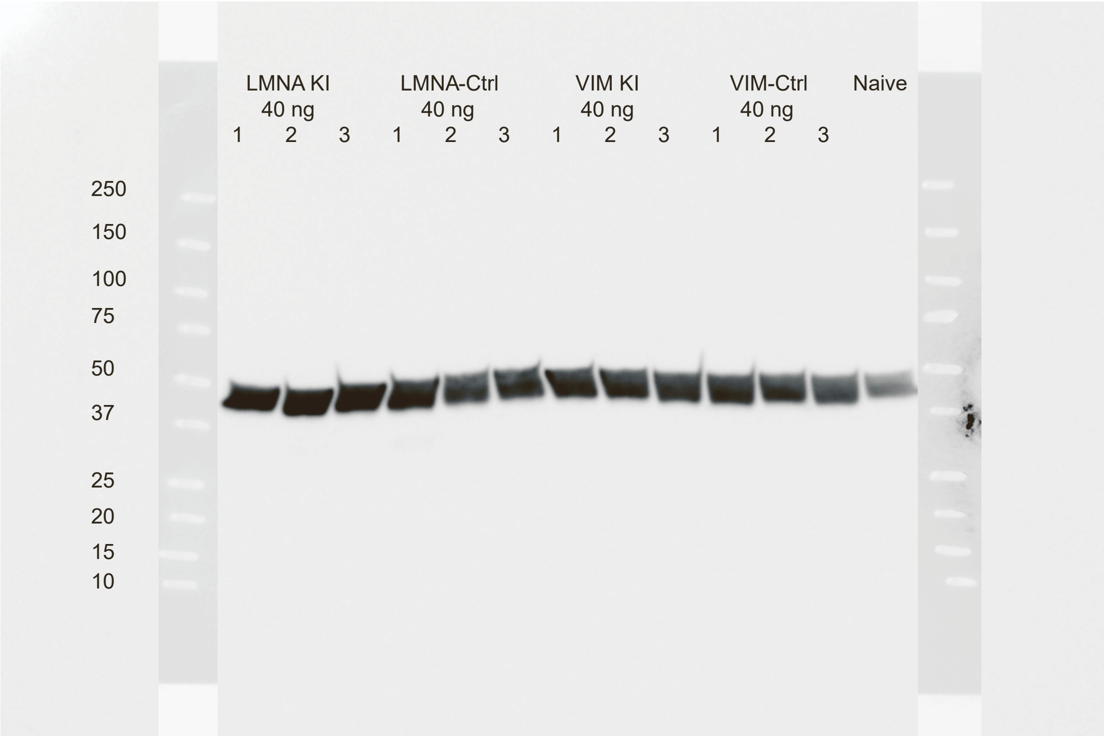

Figure S9. Uncropped western blot membranes from HeLaCas9#15 (A) Detection of Cas9 by primary antibody targeting the Flag-tag, fused to Cas9. **(B)** Detection of beta-actin, serving as a loading control.

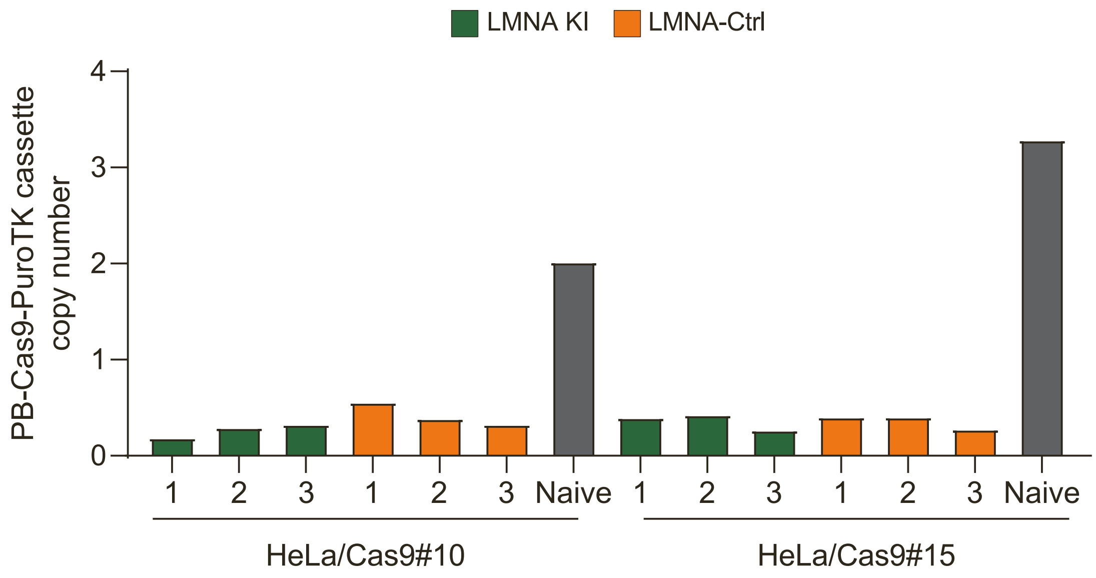

Figure S10. Copy number detection of the PBT/EFS-SpCas9-puroTK cassette in HeLa cells. (A) Detection of the PBT/EFS-SpCas9-puroTK cassette by ddPCR in HeLa/Cas9-puroTK#10 and #15 transduced with IDLV-hyPB^{Exc+}/donor and treated with FIAU.

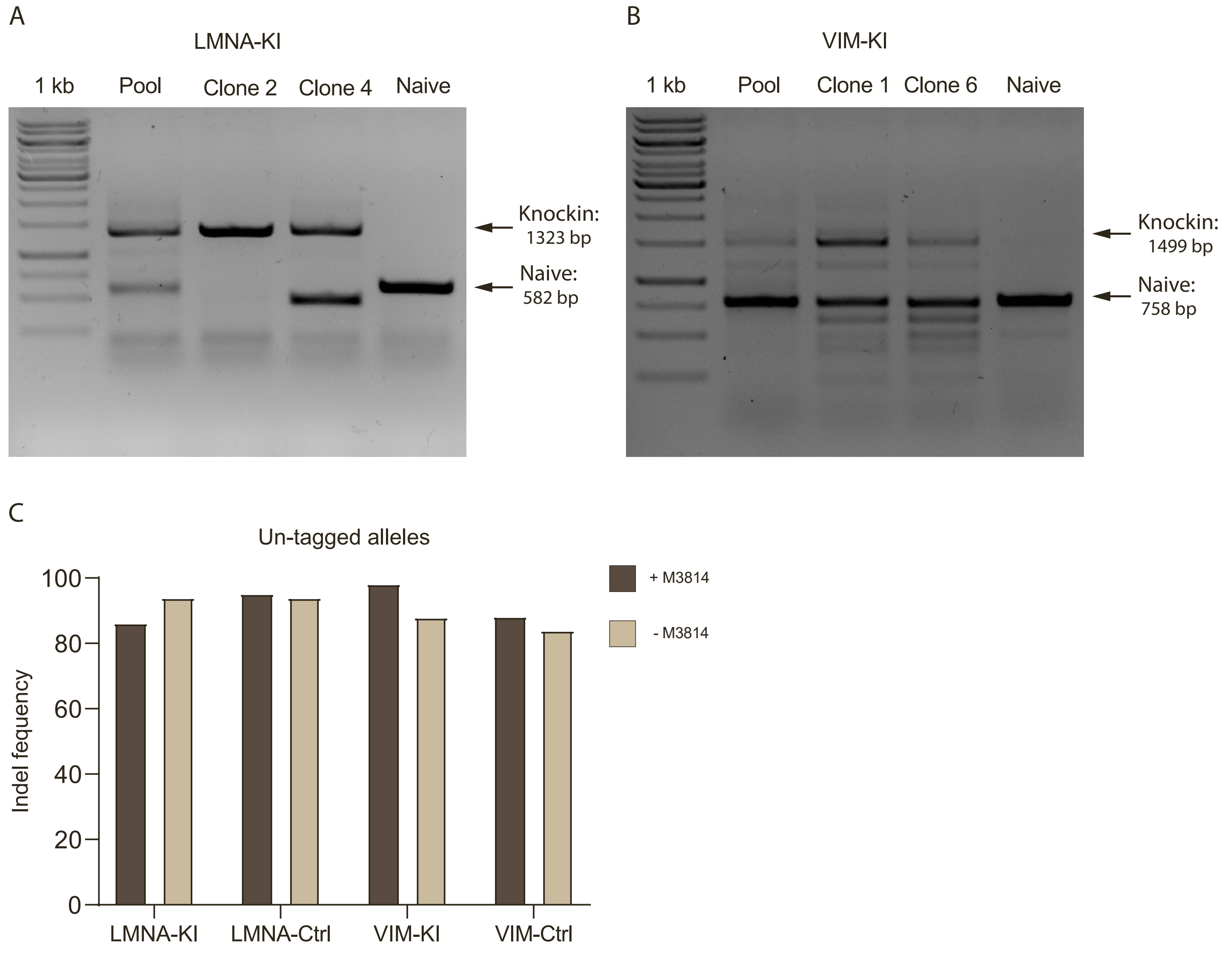

Figure S11. Validation of individual knock-in clones. (A) PCR-based validation of eGFP-tagged alleles and subsequent confirmation by sanger sequencing of *LMNA* and **(B)** *VIM*. **(C)** Indel quantification of un-tagged alleles in *LMNA* and *VIM* knock-in populations.

A

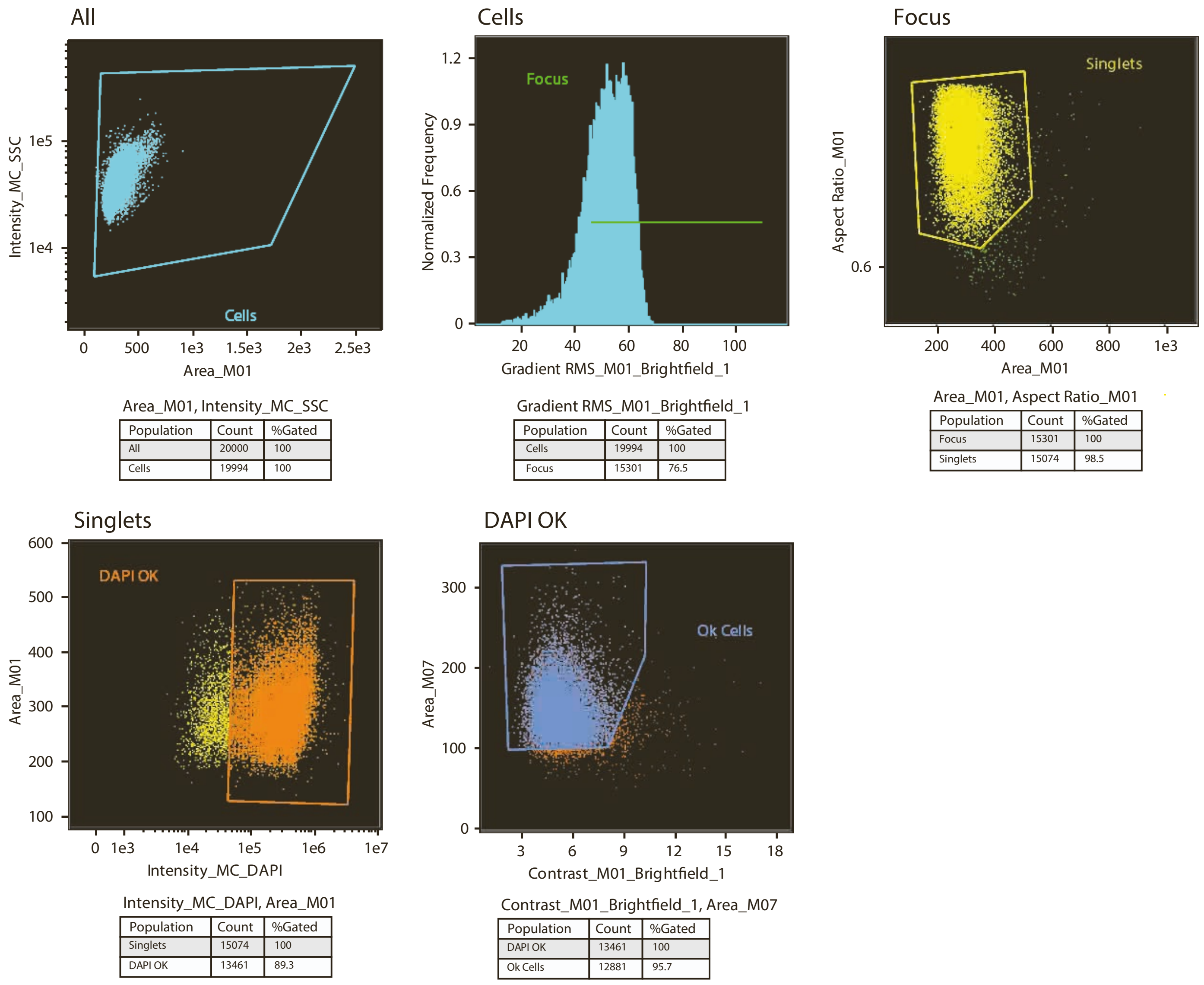

B

Wizard parameter identification

**Features**	**RD Mean(LMNA_eGFP tag.LMNA_eGFP tag)**
Bright Detail Intensity R3_MC_Ch02	2.16
Bright Detail Intensity R7_MC_Ch02	1.67
Modulation_Object(M02,Ch02,Tight)_ Ch02	1.21
Delta Centroid XY_M02_Ch02_M02_Ch02_IntensityW	0.35

Figure S12. Imagestream based quantification of correct LMNA eGFP-tagging. (A) Gating strategy used in the Imagestream analysis template applied to samples for quantification of LMNA eGFP-tagging. The last plot is an attempt to exclude cells with low viability by omitting small cells with a high contrast. **(B)** Output from the wizard used to identify the best discriminating parameters between cells with eGFP-tagged LMNA and diffuse eGFP.

Table S1. Primers and oligo sequences

**Oligo sequences used for verification of PB-transposon excision in Hek293 and HeLa SB clones**			
	Forward primer	Reverse primer	
PB-cassette excision site in the SB-transposon	ATTGACGCAAATGGGCGGTAG	GGCTGATCAGCGGGTTTAAAC	
**Oligo sequences used for detection of CRISPR-mediated DSB**			
sgRNA name	sgRNA sequence	Foward primer for ICE-analysis	Reverse primer for ICE-analysis
AFF1 sg1	CCTTCAGCTCAGTGACAGTG	AGA GGC TTG CAC TTA ACT GGT	AAA CAC TGG GCT CGT CAG TT
LMNA sg1	GCCATGGAGACCCCGTCCCAG	GGG ACT GCC CCT TTA AGA GTA	GCA AAG TTA TCG GCC TCC AG
VIM sg1	GGACCTGGTGGACATGGCTG	CGC TGA AGT AAC GGG ACC AT	CGC ACC TTG TCG ATG TAG TT
**Oligo sequences used for assesment of LMNA and VIM KI**			
	Forward primer	Reverse primer	
LMNA Locus	TGACTCAGTGTTCGCGGGAG	GGGCGAACTCACCGCGCTTT	
VIM Locus	TTATAAAAACAGCGCCCTCGG	CATGGGCGCAGCCTTACTT	
**ddPCR donor copy number**			
Albumin_forward	GCTGTCATCTCTTGTGGGCTGT		
Albumin_reverse	ACTCATGGGAGCTGCTGGTTC		
Albumin_probe	FAM-CCTGTCATGCCCACACAAATCTCTCC-BHQ		
eGFP_forward	CTGCTGCCCGACAACCAC		
eGFP_reverse	TGTGATCGCGCTTCTCGTT		
eGFP_probe	HEX-TGAGCACCCAGTCCGCCT-BHQ		
**ddPCR PB transposon copy number**			
Albumin_forward	GCTGTCATCTCTTGTGGGCTGT		
Albumin_reverse	ACTCATGGGAGCTGCTGGTTC		
Albumin_probe	HEX-CCTGTCATGCCCACACAAATCTCTCC-BHQ		
Puro_forward	CAAGAACTCTTCCTCACGCG		
Puro_reverse	GCCGATCTCGGCGAACA		
Puro_probe	FAM-ACATCGGCAAGGTGTGGGTCG-BHQ		
